# Asymmetric imaging through engineered Janus particle obscurants using a Monte Carlo approach for highly asymmetric scattering media

**DOI:** 10.1038/s41598-024-54035-7

**Published:** 2024-02-15

**Authors:** Achiles F. da Mota, Mohammad Mojtaba Sadafi, Hossein Mosallaei

**Affiliations:** 1 Department of Electrical and Computer Engineering, Northeastern University, Boston, MA 02115 USA; 2 Department of Electrical Engineering, University of Brasília (UnB), Brasília, 70910-900 Brazil

**Keywords:** Asymmetric imaging, Janus nanoparticles, Obscurants smoke, Surveillance Reconnaissance Systems, Monte Carlo ray-tracing, Scattering theory, Optics and photonics, Electrical and electronic engineering

## Abstract

The advancement of imaging systems has significantly ameliorated various technologies, including Intelligence Surveillance Reconnaissance Systems and Guidance Systems, by enhancing target detection, recognition, identification, positioning, and tracking capabilities. These systems can be countered by deploying obscurants like smoke, dust, or fog to hinder visibility and communication. However, these counter-systems affect the visibility of both sides of the cloud. In this sense, this manuscript introduces a new concept of a smoke cloud composed of engineered Janus particles to conceal the target image on one side while providing clear vision from the other. The proposed method exploits the unique scattering properties of Janus particles, which selectively interact with photons from different directions to open up the possibility of asymmetric imaging. This approach employs a model that combines a genetic algorithm with Discrete Dipole Approximation to optimize the Janus particles' geometrical parameters for the desired scattering properties. Moreover, we propose a Monte Carlo-based approach to calculate the image formed as photons pass through the cloud, considering highly asymmetric particles, such as Janus particles. The effectiveness of the cloud in disguising a target is evaluated by calculating the Probability of Detection (PD) and the Probability of Identification (PID) based on the constructed image. The optimized Janus particles can produce a cloud where it is possible to identify a target more than 50% of the time from one side (PID > 50%) while the target is not detected more than 50% of the time from the other side (PD < 50%). The results demonstrate that the Janus particle-engineered smoke enables asymmetric imaging with simultaneous concealment from one side and clear visualization from the other. This research opens intriguing possibilities for modern obscurant design and imaging systems through highly asymmetric and inhomogeneous particles besides target detection and identification capabilities in challenging environments.

## Introduction

The recent advances in imaging systems have improved the performance of several technologies, such as Intelligence Surveillance Reconnaissance Systems (SRS), Guidance Systems, and Homing Head systems^1–3^. These systems rely mostly on detecting, recognizing, identifying, positioning, and tracking a target captured by an imaging system with high accuracy and reliability, requiring a combination of advanced technologies, such as high-resolution cameras, thermal imaging sensors, radar systems, and other specialized equipment^1^. Capturing a real-time image is critical since it allows for tracking and correctly identifying targets at far distances. Moreover, another important aspect when discussing SRS is the possibility of hiding/camouflaging the target from them.

One of the most common countermeasures from SRS is using obscurants, such as smoke, dust, and fog, making it difficult or impossible for imaging systems to detect and recognize targets^1–15^. The role of obscurants is to scatter/absorb the light, thereby obstructing and distorting the image seen by the SRS camera. Thus, they can obscure the view of surveillance systems, preventing the detection and identification of targets, in addition to interfering with communication systems. Although several artificial clouds of smoke have been used over the past decades^4,16–19^, white-phosphorous-based obscurants are currently more prevalent due to their reduced toxicity^4,10^. This type of smoke is an aerosol constituted of homogeneous particles with 1–2 μm diameters that scatter light based on the Mie scattering theory^4^. According to the Mie theory^20–22^, homogeneous and radially symmetric particles scatter light in similar directions in relation to the incident angle of the incoming wave. In this sense, the current obscurant clouds hinder visualization and/or communication on both sides of the cloud, therefore also deteriorating the visibility of the individual who activated the smoke, which is undesirable for some operations.

Various methods have been proposed to address the issue of artificial smoke's impact on visualization systems, including Ligh Detection and Ranging (LiDAR) in combination with single-photon imaging systems^1,6,11–13^. These approaches aim to enhance the quality of the image rendered through the cloud, making it possible to identify the target. Another method can be seen in^23^, where the authors have used a scattering cloud serially combined with an absorbing cloud to perform asymmetric imaging. By placing an external illumination source on top of the scattering cloud (such as the sun in realistic scenarios), they achieve a good contrast difference when observing the same image from opposite sides. Using this framework, the authors can see through the cloud when observing from the absorption side and not from the scattering side.

**Figure 1:**
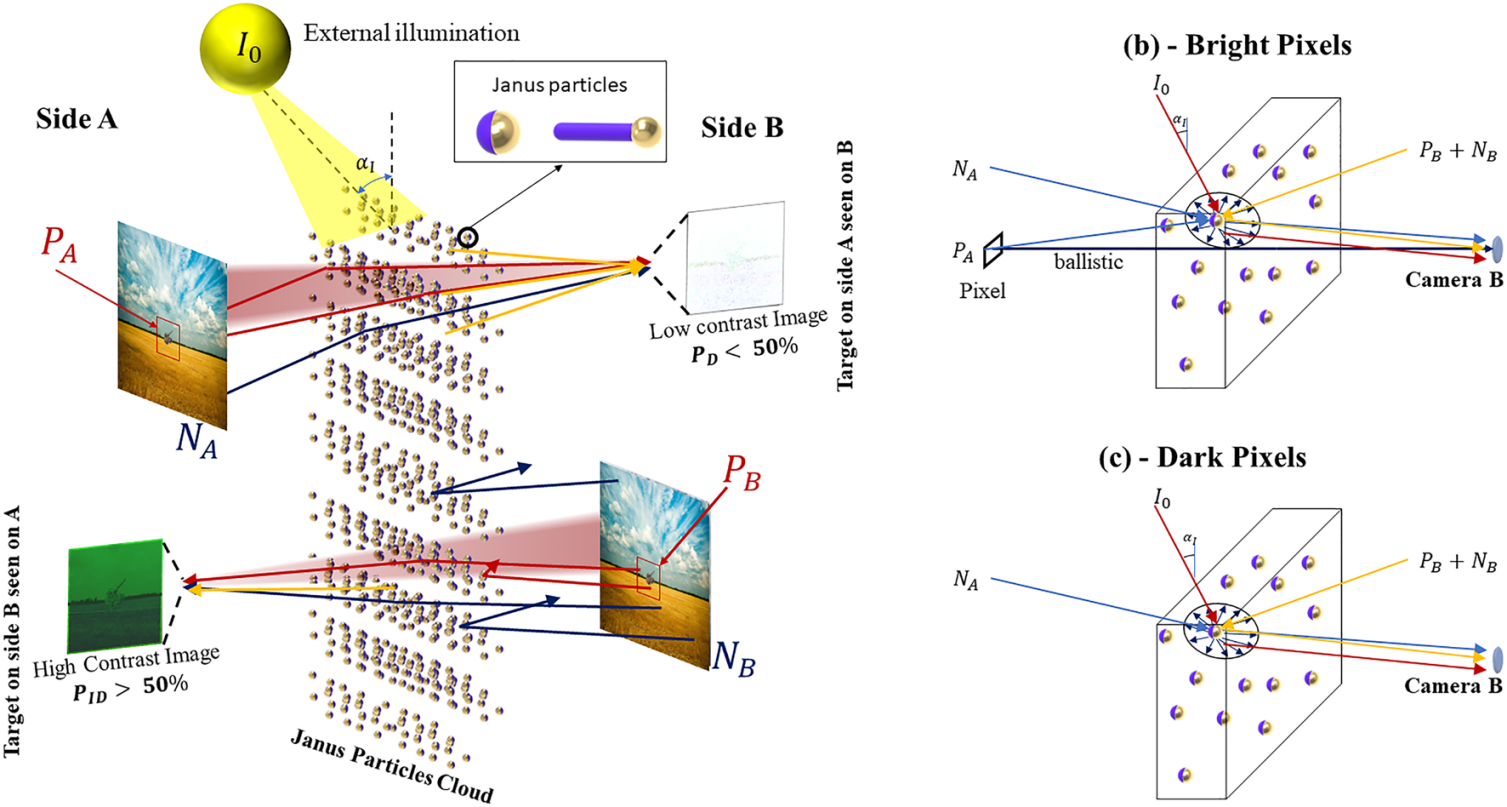
The cloud of asymmetric particles influencing the image's contrast when observed from sides B (top) and A (bottom) is shown in (**a**). In (**a**), the photons from the target *(P*_*A*_ and *P*_*B*_—red arrows), from the external illumination (yellow arrow), and from the background (NA and NB dark blue arrows) hit the cloud and get scattered. Since the cloud has a higher probability of scattering photons to the camera on side B than to A (as shown by the arrows arriving at the observer side), the system on side A (bottom) is capable of recognizing the target located on side B while the image acquired from target A on side B (top) is blurred. (**b**) and (**c**) show the proposed contrast model for studying the intensity of a bright (**b**) and dark (**c**) pixel from *P*_*A*_ illuminating camera B. In (**b**) and (**c**), in addition to the ballistic photons, the cloud also scatters noise photons from the background of A (*N*_*A*_—blue arrow), from the background of B (*N*_*B*_—yellow arrow), and an external source of light *I*_*0*_ (red arrow).

Here, we address the problem by engineering a single smoke cloud capable of portraying a useful visualization from one side while distorting and making it unrecognizable on the opposite side. This concept can be seen in Fig. 1, where a person from side A can perfectly see the target image on side B (*P*_*B*_—high contrast image), while in the opposite direction, the image gets distorted without the possibility of being recognized (*P*_*A*_—low contrast image). In this context, this study aims to design a cloud capable of increasing the contrast of the image seen on side A (*C*_*A*_) while reducing on side B (*C*_*B*_), which translates to increasing the ratio *C*_*ratio*_ = *C*_*A*_*/C*_*B*_. In the absence of a cloud, the photon radiated from a target propagates ballistically until reaching the camera, illuminating the designated pixel on the camera and consequently forming the target's image^24,25^. Nonetheless, the photons get scattered and absorbed in the presence of an obscurant cloud, reducing the number of ballistic photons (making the image darker). Moreover, the photons scattered inside the cloud change their direction, illuminating the wrong pixel on the formed image and generating noise that degrades the image quality. In this sense, one approach to designing such a system, as depicted in Fig. 1, is to generate a cloud that scatters more photons to one side of the cloud while reducing to the opposite side.

Usual spherical particles cannot scatter photons with different behavior when excited in different directions, rendering this type of particle unsuitable for the asymmetric imaging system. One fascinating approach to overcome this limitation is resorting to Janus nanoparticles. Janus particles are specialized particles with different chemical and physical properties on opposing sides^26–29^, allowing them to selectively interact with the photons differently when excited from opposing sides. These particles are considerably more complex to fabricate, but they pose as an alternative for asymmetric smoke since they can also be presented in aerosol forms^30–32^. Two common types of Janus particles are the gold-capped silica sphere and the silica-rod gold sphere matchstick, which can be synthesized using several methods, including chemical vapor deposition and electroless plating^26–28,30–58^. The asymmetry of the Janus particles makes them appropriate for controlling the scattering and absorption when excited by photons arriving from different directions. For instance, the particle may be engineered to scatter more photons to one side of the cloud with respect to the other side, increasing the number of noise photons and only degrading the image on one side of the cloud. It is worth mentioning that this property cannot be obtained using homogeneous particles. Additionally, the asymmetric properties of Janus particles also enable them to have their position and orientation actively controlled after the deployment. Numerous approaches have been proposed for controlling the position and orientation of nanoparticles, such as using electric and magnetic fields^52,59,60^, laser tweezers^61^, and ultrasound^61^, heat^54,56,57,62^, among others. This manuscript does not focus on the system that orientates the particles, which will be addressed in a future publication.

In this sense, we propose an approach to engineering Janus particles, enabling the production of asymmetric imaging. To accomplish this goal, we propose a model to calculate the impact of the cloud on the imaging system, which gives insightful information on the scattering properties of the Janus particles, which should be optimized to enable the desired functionalities. Using the inverse design approach, we combine genetic algorithm (GA) with Discrete Dipole Approximation (DDA) to optimize the geometrical parameters of the particles to achieve the desirable scattering properties.

In addition, we also propose a procedure to calculate the image formed when the photons pass through the cloud based on Monte Carlo simulation. Imaging through complex media has been a challenging task^63^. The Monte Carlo approach is a computational method commonly used to simulate the behavior of photons as they interact with materials. This approach involves generating a large number of random photon trajectories and calculating the probability of each photon interacting with the material at each step of its path. This approach helps understand the limitations of imaging systems in real-world scenarios and optimize the design of imaging systems to improve their effectiveness. Several methods have been developed to address imaging through scattering and absorbing media, including parallel processed procedures^24,25,64–80^. However, as far as the authors are aware, the procedures proposed only considered single or multi-layers of homogeneous particles, which does not represent the behavior of a Janus particle. Unlike homogenous particles, Janus particles are highly dependent on the excitation photon's direction and present different phase functions, scattering efficiency, and extinction when excited from different angles.

To address this issue, we propose a Monte Carlo approach that uses scattering theory to calculate the Point-Spread Function (PSF) and modulation transfer function (MTF) on both cloud sides considering the inhomogeneous particles. Moreover, the particles are not perfectly aligned in real scenarios, and the misalignments would instead follow probability distributions. In this sense, our method also considers the impact of the system designed to align the particles inside the cloud. Furthermore, we also include the capability to add random noise from an external illumination source to take them into account in the image constructed through the cloud.

Finally, an essential aspect of assessing the cloud’s efficiency in disguising a target is calculating the Probability of Detection (PD) and Probability of Identification (PID) of a target seen throughout the cloud^81–83^. Based on the formed image and using the theoretical eye sensibility contrast function^81,83^, we calculate the PD from side B (PD_B_) and PID from A side (PID_A_) for a target observed on each cloud side. We show that using a cloud composed of the optimized Janus particles, we can identify a target more than 50% of the times from side A (PID_A_ > 50%), while the same target is not even detected more than 50% of the times from side B (PD_B_ < 50%). Overall, this study illustrates the efficacy of the proposed cloud engineered with Janus particles, enabling asymmetric imaging with simultaneous concealment from one side and clear visualization from the other.

The manuscript is organized as follows: the “Formalism” section provides the contrast model used to understand which Janus particle properties need to be optimized to enhance asymmetric imaging. In sequence, we discuss the Monte-Carlos-based procedure to calculate the imaged formed through the asymmetric scattering cloud followed by the PID calculation. Section “Janus particle geometrical and scattering properties” demonstrates the single particle optimization using GA, and its properties. Then, section “Imaging results” provides the results of the contrast calculation and the ratio between the contrast of the image seen from both sides of the cloud. Moreover, we also study the impact of the misalignments on the contrast ratio. Finally, section “Probability of detection and identification” shows the PID results, while section “Conclusion” provides some conclusion remarks.

## Formalism

This section presents the formalism employed in this study for computing asymmetric imaging. To accomplish this task, we propose an asymmetric contrast model to understand how to optimize the geometrical parameters of the Janus particles to achieve a high contrast ratio using genetic algorithms. To evaluate the performance of the designed particles, we propose a Monte Carlo approach to calculate the contrast and the images seen on each side of the cloud. Additionally, we provide an approach to calculate PID when observed from opposite sides of the cloud.

### Asymmetric contrast model

To start our investigation on optimizing the Janus particle, we first present a model to examine how a cloud composed of asymmetric particles can influence an image's contrast when observed from opposite sides (sides A and B), as shown in Fig. 1a. In realistic circumstances, the target image (with intensity *P*_*A,B*_ where the subindices represent the side) is just a part of the scenario, where the unwanted background is assumed here as noise (with intensity *N*_*A,B*_). Moreover, we also consider an external illumination source with high-intensity *I*_*0*_ impinging on the cloud (that could represent a light source like the sun) in our model. The contrast calculation is based on how bright and dark pixels are formed in an imaging system (depicted as a camera) looking at the cloud. In the presence of the cloud, part of the bright pixels' photons passes ballistically throughout the cloud, forming the target image, while part of them gets absorbed/scattered inside the cloud, with a probability of being routed to the camera generating noise. This study aims to produce a cloud that has a higher probability of scattering photons to the camera on side B than to A, making the system on side A capable of recognizing the target located on side B and blurring the image acquired from target A on side B, as shown in Fig. 1a.

We model the contrast seen on both sides of the cloud to assess the necessary cloud scattering properties. For the sake of simplicity, we assume a camera situated on side B looking at an image on side A, and the opposite path can be obtained by simply exchanging the terms A to B in the following equations. The contrast is calculated based on the intensity of bright ($I_{A\left( B \right)}^{bright}$, where A or B denotes the side of the cloud where the camera is) and dark ($I_{A\left( B \right)}^{dark}$) pixels captured by a camera aimed at the cloud, as shown in Fig. 1b and c, respectively. As depicted in Fig. 1a, when looking from side B to a pixel on side A, the contrast $C_{B}$ can be expressed as,

1 $$C_{B} = {\frac{{I_{B}^{bright} - I_{B}^{dark} }}{{I_{B}^{bright} + I_{B}^{dark} }}}.$$

The probability of bright pixels with intensity *P*_*A*_ passing ballistically throughout the cloud is given by ${\text{e}}^{{ - \mu_{ext} L_{cloud} }}$, where $\mu_{ext}$ is the cloud extinction coefficient and $L_{cloud}$ is the length of the cloud^78^. Defining $S_{A}^{for}$ as the probability of a photon arriving at the cloud from side A being forward scattered to camera B, $S_{B}^{back}$ as the probability of a photon arriving from side B and backscattered to camera B, and $S_{B}^{up}$ as the probability of a photon arriving from the upside and scattered to camera B, we can write $I_{B}^{bright}$ as:

2 $$I_{B}^{bright} = P_{A} {\text{e}}^{{ - \mu_{ext} L_{cloud} }} + \left( {P_{A} + N_{A} } \right)S_{A}^{for} + N_{B} S_{B}^{back} + I_{0} S_{B}^{up} .$$

Note from Eq. (2) that $P_{A} {\text{e}}^{{ - \mu_{ext} L_{cloud} }}$ represents the intensity of the bright pixel in the image while $\left( {P_{A} + N_{A} } \right)S_{A}^{for}$ represents the noise photons from side A scattered to B, $\left( {N_{B} } \right)S_{B}^{back}$ is the background of B being backscattered to camera B, and $I_{0} S_{B}^{up}$ represents the noise photons from the external source. The difference between bright and dark photons is the absence of *P*_*A*_ passing ballistically through the cloud, henceforth *P*_*A*_ = 0 for dark pixels. In this sense, $I_{B}^{dark}$ can be written as

3 $$I_{B}^{dark} = \left( {P_{A} + N_{A} } \right)S_{A}^{for} + \left( {N_{B} } \right)S_{B}^{back} + I_{0} S_{B}^{up} .$$

By substituing Eqs. (3) and (2) into 1, $C_{B}$ is defined

4 $$C_{B} = \frac{{P_{A} {\text{e}}^{{ - \mu_{ext} L_{cloud} }} }}{{P_{A} {\text{e}}^{{ - \mu_{ext} L_{cloud} }} + 2\left( {P_{A} + N_{A} } \right)S_{A}^{for} + 2N_{B} S_{B}^{back} + 2I_{0} S_{B}^{up} }}.$$

As explained, the symmetry of the system allows defining the contrast of an image on side B formed in a camera on side A as,

5 $$C_{A} = \frac{{P_{B} {\text{e}}^{{ - \mu_{ext} L_{cloud} }} }}{{P_{B} {\text{e}}^{{ - \mu_{ext} L_{cloud} }} + 2\left( {P_{B} + N_{B} } \right)S_{B}^{for} + 2\left( {N_{A} } \right)S_{A}^{back} + 2I_{0} S_{A}^{up} }}.$$

The main goal of this manuscript is that an observer on side A could identify an image on side B (high $C_{A}$) while an observer on side B would not identify the image on side A (low $C_{B}$). To assess the performance of the cloud, we define the figure of merit contrast ratio

6 $$C_{ratio} = \frac{{C_{A} }}{{{{C}}_{{{B}}} }} = \frac{{P_{B} {\text{e}}^{{ - \mu_{ext} L_{cloud} }} }}{{P_{A} {\text{e}}^{{ - \mu_{ext} L_{cloud} }} }} \times \frac{{P_{A} {\text{e}}^{{ - \mu_{ext} L_{cloud} }} + 2\left( {P_{A} + N_{A} } \right)S_{A}^{for} + 2N_{B} S_{B}^{back} + 2I_{0} S_{B}^{up} }}{{P_{B} {\text{e}}^{{ - \mu_{ext} L_{cloud} }} + 2\left( {P_{B} + N_{B} } \right)S_{B}^{for} + 2N_{A} S_{A}^{back} + 2I_{0} S_{A}^{up} }},$$

where the purpose is to maximize $C_{ratio}$. Based on Eq. (6), the critical element to enhance the contrast ratio is the cloud's ability to steer the noise photons to B side (maximize $S_{A}^{for}$, $S_{B}^{for}$, $S_{B}^{up}$) while reducing to A side (minimizing $S_{B}^{for} ,S_{A}^{back} ,S_{A}^{up}$). One critical consideration is that $\mu_{ext}$ is the same for a photon propagating in both directions inside the cloud (explained by the reciprocity theorem—proof in Supplementary information), rendering the same ballistic attenuation on both sides of the cloud.

The next step is connecting $S_{A,B}^{for}$, $S_{A,B}^{for}$, $S_{A,B}^{up}$ with single-particle properties. To accomplish that, we assume that the photon collides with particles inside the cloud with an incident elevation and azimuthal angles $\theta_{i}$ and $\varphi_{i}$, respectively. Given the inhomogeneous properties of the Janus particles, the incident angle has a massive impact on the particles' scattering properties. Moreover, although there have been analytical approaches to calculate the scattering parameters of metallic-coated dielectric spheres^84^, we opted for using the Direct Dipole Approximation (DDA)^85–88^ since it is a numerical approach which can employed for any geometry of Janus particles (full details in the Supplementary Information). The probability of a photon arriving at the particle with incident angles ($\theta_{i}$,$\varphi_{i}$) and being scattered to ($\theta$,$\varphi$) is proportional to the particle gain $G\left( {\theta_{i} , \varphi_{i} ,\theta ,\varphi } \right) = \eta_{rad} \left( {\theta_{i} , \varphi_{i} } \right)D\left( {\theta_{i} , \varphi_{i} ,\theta ,\varphi } \right)$, where $\eta_{rad} \left( {\theta_{i} , \varphi_{i} } \right) = \sigma_{sct} \left( {\theta_{i} , \varphi_{i} } \right)/\sigma_{ext} \left( {\theta_{i} , \varphi_{i} } \right)$ is the radiation efficiency, $\sigma_{sct}$ and $\sigma_{ext}$ are the scattering and extinction cross-sections, and $D$ is the particle's directivity (note *D* is also known as the scattering phase function). Since $D/4\pi$ can be interpreted as a probability density function, we define $S_{A,B}^{for}$, $S_{A,B}^{back}$, $S_{A,B}^{up}$ as

7 $$S_{A\left( B \right)}^{for} \left( {\theta_{i} , \varphi_{i} } \right) = \frac{1}{4\pi }\mathop \smallint \limits_{{0\left( {3\pi /4} \right)}}^{{\frac{\pi }{4}\left( \pi \right)}} {\text{d}}\theta \mathop \smallint \limits_{0}^{2\pi } G\left( {0\left( \pi \right),0,\theta ,\varphi } \right)\sin {\theta d}\varphi ,$$

8 $$S_{A\left( B \right)}^{back} \left( {\theta_{i} , \varphi_{i} } \right) = \frac{1}{4\pi }\mathop \smallint \limits_{{\frac{3\pi }{4}\left( 0 \right)}}^{{\pi \left( {\frac{\pi }{4}} \right)}} {\text{d}}\theta \mathop \smallint \limits_{0}^{2\pi } G\left( {0\left( \pi \right), 0,\theta ,\varphi } \right)\sin {\theta d}\varphi ,$$

9 $$S_{A\left( B \right)}^{up} \left( {\theta_{i} , \varphi_{i} } \right) = \frac{1}{4\pi }\mathop \smallint \limits_{{\frac{3\pi }{4}\left( 0 \right)}}^{{\pi \left( {\frac{\pi }{4}} \right)}} {\text{d}}\theta \mathop \smallint \limits_{0}^{2\pi } G\left( {\frac{\pi }{2} + \alpha_{I} , 0,\theta ,\varphi } \right)\sin \theta {\text{d}}\varphi .$$

Note from Eqs. (7)–(9) that we consider parallel or perpendicular incidence upon the particles since the goal is to define equations to relate the cloud to the single particle's properties. We would like to add that optimizing the particle's considering multiple incidences is also possible, but it would considerably increase the computational effort. Therefore we focused our approach displayed in (7)–(9).

**Figure 2:**
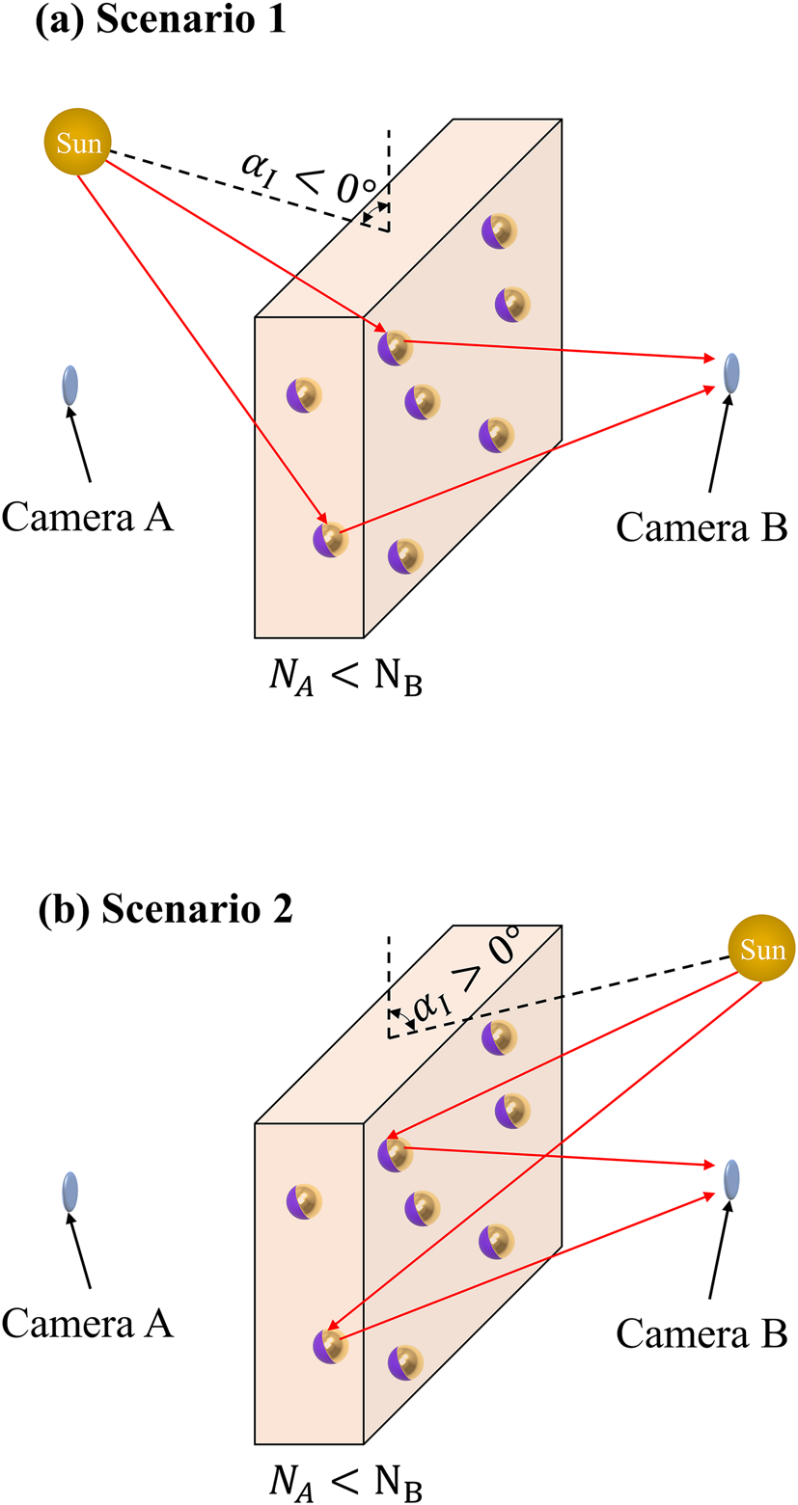
Proposed scenarios where the primary source of noise photons is located at an angle ${\upalpha }_{{\text{I}}}$ from the cloud, where $\alpha_{I} = 0^\circ$ correspond to the source being atop the cloud. The two scenarios are defined when the primary source moves from $\alpha_{I} = 0^\circ$ to side A, rendering $\alpha_{I} < 0^\circ$ (**a**), and to side B, rendering $\alpha_{I} \ge 0^\circ$ (**b**).

Once the correlation between the single-particle scattering properties and the cloud performance for asymmetric imaging has been established, the final step is optimizing the single-particle geometrical properties to increase *C*_*ratio*_. In realistic scenarios, the primary source of noise photons during the day is the sun, which can be located on sides A or B, as shown in Fig. 2a and b, respectively. Each scenario demands different particle scattering properties in order to increase *C*_*ratio*_. In scenario 1 (Fig. 2a), where the sun is on side A and *N*_*A*_ > *N*_*B*_, we need to increase the noise on camera B by maximizing the transmission of photons from A to B ($S_{A}^{for}$) while reducing the reflection from side A $\left( {S_{A}^{back} } \right)$. In mathematical terms, this represents

10 $${\text{maximize}}\left\{ {\frac{{S_{A}^{for} }}{{S_{A}^{back} }}} \right\}.$$

To increase *C*_*ratio*_ in scenario 2 (where the sun is on side B and *N*_*B*_ > *N*_*A*_), we need to maximize the reflection of photons on side B ($S_{B}^{back}$), while reducing the transmission of scattered photons from side B to A ($S_{B}^{for}$). In this sense,

11 $${\text{maximize}}\left\{ {\frac{{S_{B}^{back} }}{{S_{B}^{for} }}} \right\}.$$

Using the two proposed scenarios, we can design a set of particles capable of producing asymmetric imaging in any scenario with a primary source of the noise of photons.

### Monte Carlo model

Monte Carlo (MC) simulations have proven to be valuable for modeling and simulating light propagation through scattering media, such as biological tissue or clouds^24,25,65–80^. In the context of imaging through scattering media, Monte Carlo simulations are used to model the scattering and absorption of light and its detection by a sensor or camera. In a typical Monte Carlo simulation, the scattering properties of the medium are first characterized and modeled. This information is later used to simulate light propagation through the medium, which involves the generation of many random photons paths. The paths are modeled based on probability distributions that account for the scattering and absorption of light by the medium and help predict the intensity and distribution of light that reaches a sensor or camera, as well as the noise and other artifacts that might be present in the resulting image. The method relies upon sending many photons to the scattering medium and, based on the probability properties of the scatters, computing the propagation path until the receptor.

**Figure 3:**
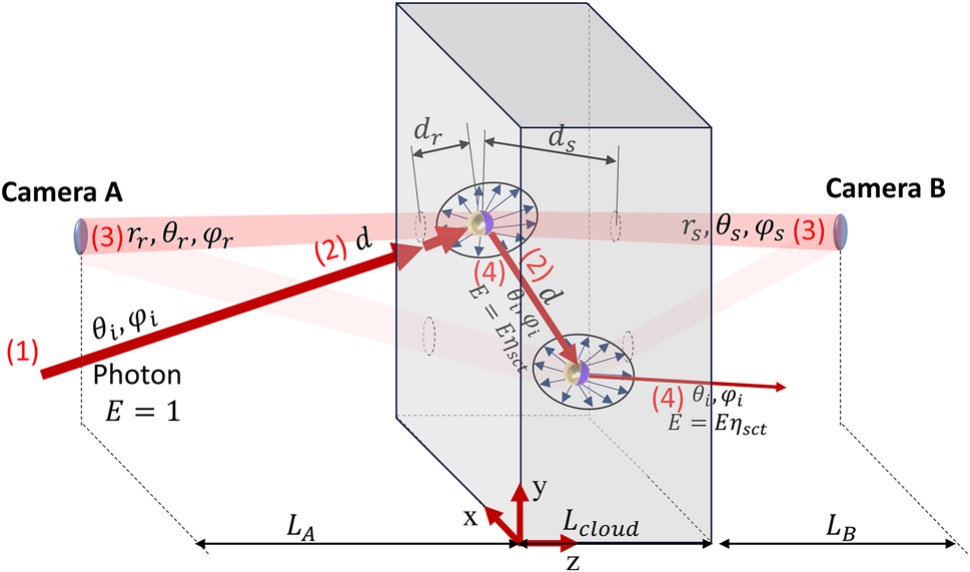
Schematic of the ray tracing approach based on Monte Carlo simulation. The photon is created (1) with *E* = 1 at random direction (*θ*_*i*_,*φ*_*i*_) and propagates without obstacles to the cloud. After arriving at the cloud, the photon propagates a random distance *d* randomly generated based on $\mu_{exc} \left( {\theta_{i} + \Delta \theta , \varphi_{i} + \Delta \varphi } \right)$ (2). After propagating to the scattering event, we calculate the probability of this photon being scattered to camera A or B (3), followed by the determination of the photon's new direction (*θ*_*i*_,*φ*_*i*_) based on $D\left( {\theta_{i} + \Delta \theta , \varphi_{i} + \Delta \varphi ,\theta , \varphi } \right)$ and energy $E = E\eta_{rad}$ (4). The steps (2)–(4) are repeated until $E \le \eta$.

The procedure starts by generating a photon with intensity *E* = 1 on side A propagating with angle (*θ*_*i*_,*φ*_*i*_), as shown in Fig. 3 (step 1). To reduce the computational time, we limit the region where the photons may exist based on cameras A's and B's numerical aperture (*NA*). The numerical aperture defines the maximum acceptance angle $\left( {\alpha_{max} = \tan^{ - 1} \left[ {\left( {L_{A} /L_{B} } \right)\tan \left( {{\text{asin}}\left( {NA} \right)} \right)} \right]} \right)$, where the photons outside the acceptance region cannot be scattered and sensed by the camera and henceforth are not considered in our model. Here, our scatters are highly asymmetrical, and $\mu_{exc}$ is dependent on $\left( {\theta_{i} , \varphi_{i} } \right)$. In addition, we also consider that the particles inside the cloud could also present some disorientation since they suffer from several external forces that act against the alignment system. To take this effect into account, we consider an elevation and azimuthal disorientation (Δ*θ*, Δ*φ*, respectively) governed by a normal distribution with variance *σ*_*θ*_ and *σ*_*φ*_, respectively. Therefore, the distance where the photon propagates inside the cloud (*d*) until a scattering/absorption event is randomly generated by (step 2):

12 $$d = - \frac{\log \left( f \right)}{{\mu_{ext} \left( {\theta_{i} + \Delta \theta , \varphi_{i} + \Delta \varphi } \right)}},$$

where *f* is a random number between 0 and 1. After propagating a distance *d*, if the photon is still inside the acceptance region, we calculate the probability of this photon being scattered to the camera on B and A side by using the relation (step 3),

13 $$Pr_{B} \left( {\theta_{i} , \varphi_{i} ,\theta_{s} , \varphi_{s} ,r_{s} ,d_{s} } \right) = \eta_{rad} \frac{{D\left( {\theta_{i} + \Delta \theta , \varphi_{i} + \Delta \varphi ,\theta_{s} , \varphi_{s} } \right)}}{{4\pi r_{s}^{2} }}A_{ape} P_{pass} \left( {\theta_{s} , \varphi_{s} ,d_{s} } \right),$$

14 $$Pr_{A} \left( {\theta_{i} , \varphi_{i} ,\theta_{s} , \varphi_{s} ,r_{s} ,d_{s} } \right) = \eta_{rad} \frac{{D\left( {\theta_{i} + \Delta \theta , \varphi_{i} + \Delta \varphi ,\theta_{r} , \varphi_{r} } \right)}}{{4\pi r_{r}^{2} }}A_{ape} P_{pass} \left( {\theta_{r} , \varphi_{r} ,d_{r} } \right),$$

where $\left( {r_{s\left( r \right)} ,\theta_{s\left( r \right)} , \varphi_{s\left( r \right)} } \right)$ are distance, the azimuthal and elevation angle from the scattering event to camera B(A) (see Fig. 3), *A*_*ape*_ is the aperture area, $\eta_{rad} = \frac{{\mu_{sct} \left( {\theta_{i} + \Delta \theta , \varphi_{i} + \Delta \varphi } \right)}}{{\mu_{ext} \left( {\theta_{i} + \Delta \theta , \varphi_{i} + \Delta \varphi } \right)}}$ is the probability of the photon being radiated instead of absorbed, $P_{pass} \left( {\theta_{s} , \varphi_{s} ,d_{s} } \right) = {\text{e}}^{{ - \mu_{exc} \left( {\theta_{s} , \varphi_{s} } \right)d_{s} }}$ is the probability of the photon passing through the remaining length of the cloud (*d*_*s*_) without a scattering/absorption event. The directivity pattern represents the probability of a photon being scattered in a specific direction concerning an isotropic source. Therefore, the term $\frac{{D\left( {\theta_{i} + \Delta \theta , \varphi_{i} + \Delta \varphi ,\theta_{s} , \varphi_{s} } \right)}}{{4\pi r_{s}^{2} }}A_{ape}$ represents the probability of the photon arriving at a misaligned particle ($\Delta \theta , \Delta \varphi$) from directions $\left( {\theta_{i} , \varphi_{i} } \right)$ and being scattered in the specific path to camera B ($\theta_{s} , \varphi_{s}$) or A ($\theta_{r} , \varphi_{r}$). After calculating the probability, we find the intensity of the photon that arrives at camera B ($I_{cam}^{B}$) and A ($I_{cam}^{A}$) as,

15 $$I_{cam}^{B} \left( {i, j} \right) = I_{cam}^{B} \left( {i, j} \right) + EPr_{B} \left( {\theta_{i} , \varphi_{i} ,\theta_{s} , \varphi_{s} ,d_{s} } \right),$$

16 $$I_{cam}^{A} \left( {i, j} \right) = I_{cam}^{A} \left( {i, j} \right) + EPr_{A} \left( {\theta_{i} , \varphi_{i} ,\theta_{r} , \varphi_{r} ,d_{r} } \right).$$

To position the $I_{cam}^{B\left( A \right)} \left( {i, j} \right)$, we consider a charge-coupled device (CCD) with *N*_*pix*_ × *N*_*pix*_ pixels and size *l* placed on the focus of a biconvex lens. Note that Eqs. (15) and (16) are written as pseudo-code to highlight that we need to accumulate the energy of the photons at the CCD as we propagate them. The indices *i* and *j* are the horizontal and verticalpixel indices of the camera, respectively, calculated using raytracing matrices^64^. After arranging $I_{cam}^{B\left( A \right)} \left( {i, j} \right)$, the photon gets scattered into a new direction $\theta_{i} , \varphi_{i}$ (randomly generated based on $D\left( {\theta_{i} + \Delta \theta , \varphi_{i} + \Delta \varphi ,\theta , \varphi } \right)$), and energy is updated $E = E\eta_{rad}$ (step 4). This procedure repeats until the photon energy is lower than an energy threshold *ζ*. Moreover, the whole procedure is realized for *N*_*photon*s_, where higher *N*_*photon*s_ provide higher simulation accuracy but increase the computational time.

**Figure 4:**
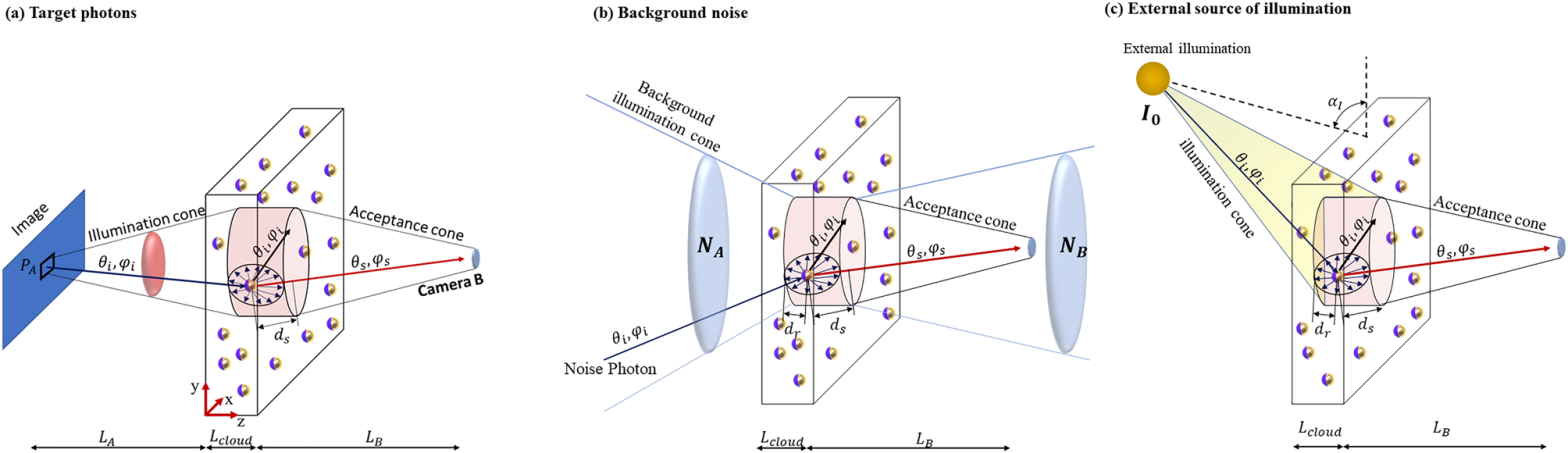
Initial conditions for the photons propagating inside the cloud. (**a**) Shows the photons arriving at the cloud from the target (*P*_*A*_), and the initial propagation angles are randomly chosen at the interval $\left( {0,0} \right) \le \left( {\theta_{i} ,\varphi_{i} } \right) \le \left( {\alpha_{max} ,2\pi } \right) .$ The initial directions for *N*_*A(B)*_, shown in (**b**) are uniformly distributed inside the interval defined by $0\left( {\pi /2} \right) \le \theta_{i} \le \pi /2\left( \pi \right) ;0 \le \varphi_{i} \le 2\pi$, while the initial positions *x*_*i*_ and *y*_*i*_ are uniformly distributed inside the acceptance circle at $z_{i} = 0\left( {L_{cloud} } \right)$. Finally, (**c**) illustrate the initial conditions for the photons arriving from I_0_, where $\theta_{i} = \frac{\pi }{2} - \alpha_{I} ,\varphi_{i} = 3\pi /2$, and the initial positions *x*_*i*_ and *y*_*i*_ are uniformly distributed inside the acceptance cylinder top and frontal face.

Equations (12)–(16) explain how the Monte Carlo approach works inside the scattering cloud, but the initial conditions for the photon arriving depend on the illumination source. In this sense, the initial photons arriving from the target (*P*_*A*_) have propagation angles randomly chosen at the interval $\left( {0,0} \right) \le \left( {\theta_{i} ,\varphi_{i} } \right) \le \left( {\alpha_{max} ,2\pi } \right)$, and they propagate uninterrupted until arriving at the cloud, as seen in Fig. 4a. Moreover, for a photon arriving from the target at B side, the propagation angle is randomly chosen at the interval $\left( {\pi - \alpha_{max} ,0} \right) \le \left( {\theta_{i} ,\varphi_{i} } \right) \le \left( {\pi ,2\pi } \right)$. To consider the background noise from sides A (*N*_*A*_) and B (*N*_*B*_), the procedure is repeated with different initial conditions, as illustrated in Fig. 4b. In this scenario, the initial directions for *N*_*A*_ are uniformly distributed inside the interval defined by $0 \le \theta_{i} \le \pi /2 ;0 \le \varphi_{i} \le 2\pi$, while the initial positions *x*_*i*_ and *y*_*i*_ are uniformly distributed inside the acceptance circle at $z_{i} = 0$. On the other hand, for *N*_*B*_, the initial directions are distributed inside the interval defined by $\pi /2 \le \theta_{i} \le \pi ;0 \le \varphi_{i} \le 2\pi$, while the initial positions *x*_*i*_ and *y*_*i*_ are uniformly distributed inside the acceptance circle at $z_{i} = L_{cloud}$. Finally, for the external illumination impinging on the structure at an angle *α*_*I*_ (as seen in Fig. S5 (c)), the initial condition for the *I*_*0*_ are $\theta_{i} = \frac{\pi }{2} - \alpha_{I} ,\varphi_{i} = 3\pi /2$ (note that the choice of $\varphi_{i}$ do not impact the results since the particles present rotational symmetry), and the initial positions *x*_*i*_ and *y*_*i*_ are uniformly distributed inside the acceptance cylinder top and frontal face (see Fig. 4c), where the front face is defined as $z_{i} = 0 {\text{or}} L_{cloud}$ for *α*_*I*_ < 0 or* α*_*I*_ > 0, respectively. After performing the photon propagation, the intensity for each noise source is computed to study their impact on the contrast. The intensity map generated on side A(B) by *P*_*B*_ ($I_{cam}^{A\left( B \right)} \left( {i, j} \right)$), *N*_*A*_($I_{{N_{A} }}^{A\left( B \right)} \left( {i, j} \right)$), *N*_*B*_($I_{{N_{B} }}^{A\left( B \right)} \left( {i, j} \right)$)_*,*_ and *I*_*0*_ ($I_{{I_{0} }}^{A\left( B \right)} \left( {i, j} \right)$) follows the same procedure presented on Eqs. (15 and 16).

Note that a different Monte Carlo simulation is performed for each noise source since the initial conditions drastically change. It is essential to highlight that all intensity maps $I_{cam}^{B\left( A \right)}$, $I_{{N_{A} }}^{B\left( A \right)}$, $I_{{N_{B} }}^{B\left( A \right)}$ and $I_{{I_{0} }}^{B\left( A \right)}$ are calculated using the same number of generated photons for comparison reasons, allowing individual control of each noise source's power. After finalizing propagating all photons and obtaining the $I_{cam}^{B\left( A \right)}$, $I_{{N_{A} }}^{B\left( A \right)}$, $I_{{N_{B} }}^{B\left( A \right)}$ and $I_{{I_{0} }}^{B\left( A \right)}$, the next step is to determine the point-spread function from the image in A captured by a camera in B ($PSF_{B}$) as

17 $$PSF_{B} \left( {i, j} \right) = P_{A} I_{{P_{A} }}^{B} \left( {i, j} \right) + N_{A} I_{{N_{A} }}^{B} \left( {i, j} \right) + N_{B} I_{{N_{B} }}^{B} \left( {i, j} \right) + I_{0} I_{{I_{0} }}^{B} \left( {i, j} \right),$$

where $P_{A}$ is the pixel intensity, $N_{A}$ and $N_{B}$ are the background noise intensities and $I_{0}$ is the intensity of the external source of light. When assuming that *I*_*0*_ is responsible for illuminating the target and the cloud, a relation between them can be built and is shown in detail in the Supplementary information. The modulation transfer function from the image in A captured by a camera in B ($MTF_{B}$) is given by the 2-dimensional Fourier transform (${\mathcal{F}}^{2D}$) of $PSF_{B} \left( {i, j} \right)$ divided by the maximum of the function, or

18 $$MTF_{B} \left( {k_{i} ,k_{j} } \right) = \frac{{{\mathcal{F}}^{2D} \left\{ {PSF_{B} \left( {i, j} \right)} \right\}}}{{\max \left( {{\mathcal{F}}^{2D} \left\{ {PSF_{B} \left( {i, j} \right)} \right\}} \right)}}.$$

The MTF is a 2D function that provides information on the contrast calculated for all spatial frequencies ($k_{i} ,k_{j}$). In this sense, to quantify the image contrast, we define $C_{B}$ as the average of $MTF_{B}$ minus the DC component of $MTF_{B} \left( {0,0} \right)$, as follows,

19 $$C_{B} = \left\langle {MTF_{B} } \right\rangle = \frac{{\mathop \sum \nolimits_{{i,j \ne 0}}^{{}} MTF_{B} \left( {k_{i} ,k_{j} } \right)}}{{N_{{pix}}^{2} }}.$$

Finally, the ratio between the contrasts ($C_{ratio}$) when the image is seen from both sides of the cloud is given by (note the *C*_*A*_ is calculated using the same procedure described for *C*_*B*_),

20 $$C_{ratio} = \frac{{C_{A} }}{{C_{B} }}$$

The final step to analyze the asymmetric imaging is to compute the observed image after propagating through the cloud on side B (A) ($O_{propag}^{B\left( A \right)} \left( {i, j} \right)$), which can be calculated as

21 $$O_{propag}^{B\left( A \right)} \left( {i, j} \right) = P_{A\left( B \right)} \left[ {I_{{P_{A\left( B \right)} }}^{B\left( A \right)} \left( {i, j} \right)} \right]*O\left( {i, j} \right) + N_{pix}^{2} \left( {N_{A} I_{{N_{A} }}^{B\left( A \right)} \left( {i, j} \right) + N_{B} I_{{N_{B} }}^{B\left( A \right)} \left( {i, j} \right) + I_{0} I_{ilu}^{B\left( A \right)} \left( {i, j} \right)} \right)$$

where $O\left( {i, j} \right)$ is the original image. Note from (23) that the image is formed by the convolution of $I_{{P_{A} }}^{B} \left( {i, j} \right)$ with the original image $O\left( {i, j} \right)$ added with the noise photons.

### Probability of Detection (PD) and Probability of Identification (PID)

After obtaining the MTFs from both sides of the cloud, we may use the formalism proposed in^81^ to calculate the PID of a target. The model works based on contrast theory, and each frequency harmonic (*k*_*i*_ and *k*_*j*_) of the image in the frequency domain (Fourier transformed) is called "cues" to identify the target. The more frequencies are correctly identified by the observer, the more likely the observer is to identify the target correctly. In this sense, the minimum of contrast required by the "naked eye" to identify that "cue" is defined as the Contrast threshold function (CTF_eye_)^83^. However, CTF_eye_ needs to be corrected since the photons pass through the cloud, which interferes with the contrast threshold, and this correction is performed by defining CTF_system_ = CTF_eye_/MTF^81^. Then we integrate all the cues where the image's contrast (*C*_*tgt*_) of the target is higher than the contrast threshold of the system ($\delta \left( {\frac{{C_{tgt} \left( {R_{tgt} ,\xi ,\psi } \right)}}{{CTF_{sys} \left( {\xi ,\psi } \right)}}} \right)$), to calculate the target task performance (TTP) metric ${\Phi }$  ^81^,

22 $${\Phi } = \left\{ {\mathop \smallint \limits_{ - \infty }^{\infty } \mathop \smallint \limits_{ - \infty }^{\infty } \frac{1}{{R_{tgt}^{2} }}\left[ {\frac{{C_{tgt} \left( {R_{tgt} ,\xi ,\psi } \right)}}{{CTF_{sys} \left( {\xi ,\psi } \right)}}} \right]\delta \left( {\frac{{C_{tgt} \left( {R_{tgt} ,\xi ,\psi } \right)}}{{CTF_{sys} \left( {\xi ,\psi } \right)}}} \right){\text{d}}\xi {\text{d}}\psi } \right\}^{1/2} ,$$

where *R*_*tgt*_ is the distance of the observer to the target (in km), and $\xi ,\psi$ are the spatial frequency in cycles per milliradians (mrad^−1^). Note that $\xi ,\psi$ are directly related *k*_*i*_,*k*_*j*_, and can be manipulated to simulate a target moving farther or closer to the observer. Based on Φ, we use the error function (erf) to calculate the PID as^81^,

23 $${\text{PID}} = {\text{erf}}\left( {{\Phi }/\left( {{\text{V}}_{84} *L_{tgt} } \right)} \right),$$

where *V*_*84*_ = 2.08*V*_*50*_, *V*_*50*_ is the "cycles on target" required to correctly identify the target 50% of the time, and *L*_*tgt*_ is the target size in meters^81^. The probability of detection (PD) is calculated using Eq. (25) corisering a different *V*_*50*_. It is crucial to emphasize that *V*_*50*_ is experimentally obtained and varies for different targets. The goal of this formulation is to assess how the cloud may affect the PID of an observer on side A looking at a target on side B (PID_A_) and an observer on side B looking at a target on side A (PID_B_).

## Janus particle geometrical and scattering properties

**Figure 5:**
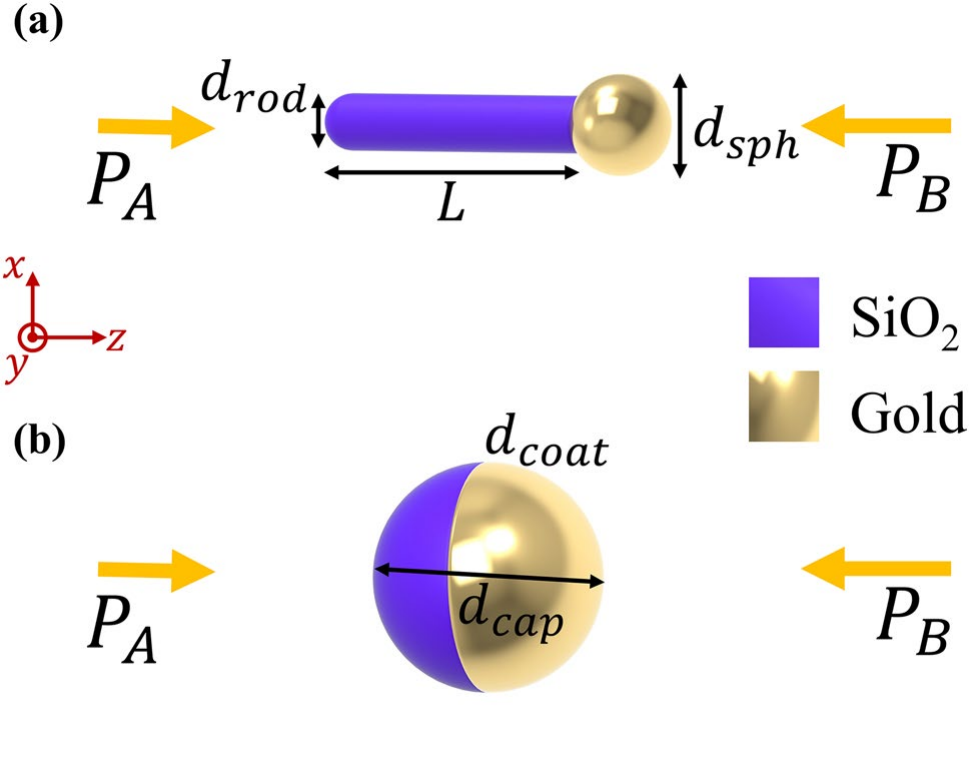
Two particles used for asymmetric imaging: (**a**) shows a matchstick consisting of a silica rod (blue) with length *L* and diameter *d*_*rod*_ connected to a gold sphere (golden) with diameter *d*_*sph*_ and (**b**) shows a silica sphere with diameter *d*_*cap*_ half-coated by a *d*_*coat*_ thick gold layer.

After the formalism, we proceed to engineer the particles for two distinct scenarios, considering two commonly used asymmetric particle configurations: silica-rod gold sphere matchstick (depicted in Fig. 5a) and the gold-capped silica sphere (depicted in Fig. 5b). The matchstick consists of a rod with length *L* and diameter *d*_*rod*_ connected to a gold sphere with diameter *d*_*sph*_, while the capped silica consists of a sphere with a diameter *d*_*cap*_ half-coated by a *d*_*coat*_ thick gold layer. By implementing Eqs. (10–11) as the objective function in a genetic algorithm (GA), the geometrical properties of the particles have been optimized for each scenario. Moreover, the operation wavelength (*λ*) is also optimized by the GA (constraining the interval to 400–800 nm) to achieve the highest possible contrast. A detailed description of how to calculate $S_{A,B}^{for}$, $S_{A,B}^{for}$, using the DDA is provided in the Supplementary information.

**Figure 6:**
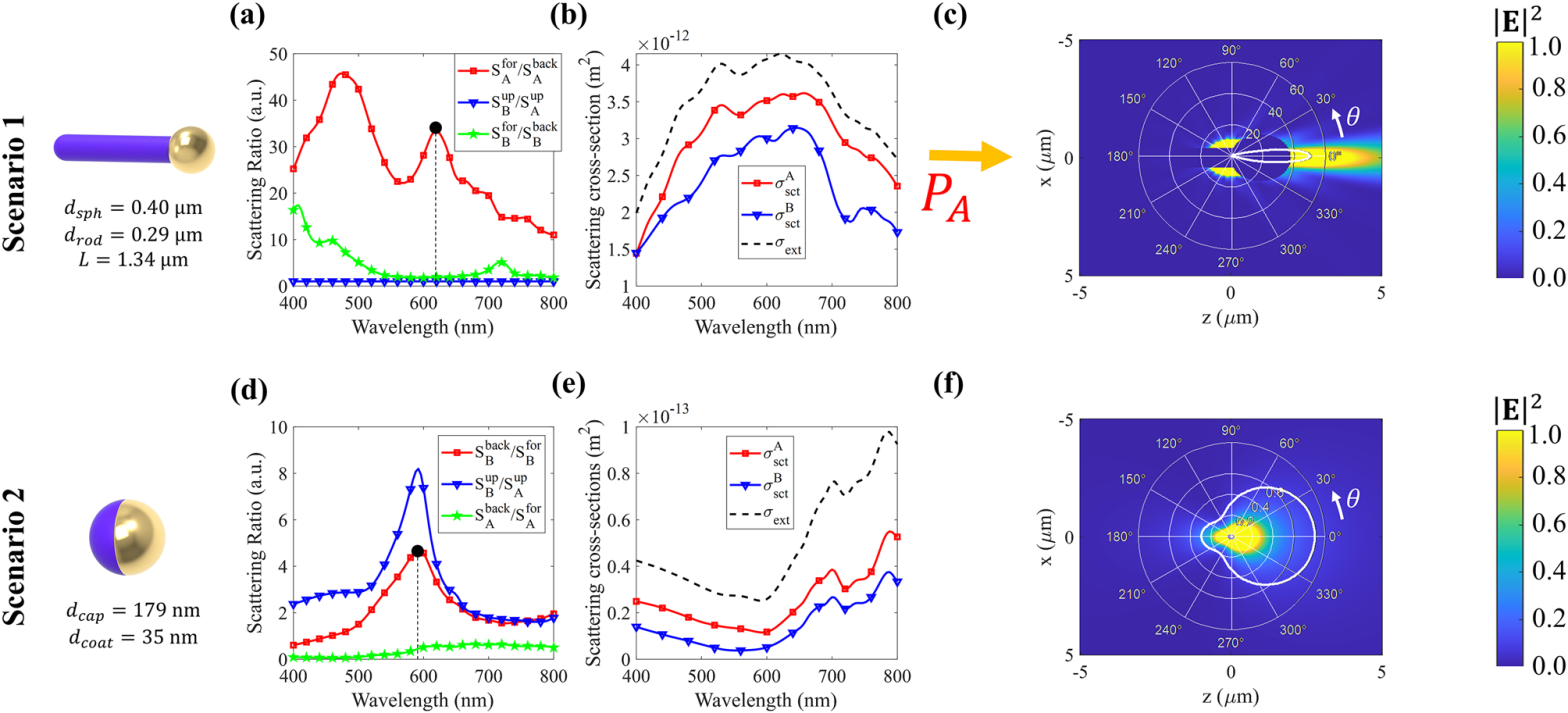
Optimized particles geometrical parameters and properties for the three proposed scenarios. For scenario 1, $S_{A}^{for} /S_{A}^{back}$ (red line with circles), $S_{B}^{up} /S_{A}^{up}$ (blue line with triangles) $S_{B}^{for} /S_{B}^{back}$ (green line with stars) are shown in (**a**) while $\sigma_{sct}^{A}$(red line with squares), $\sigma_{sct}^{B}$ (blue dashed line with triangles) and $\sigma_{ext}$ (black dashed line) are in (**b**). For scenario 2, $S_{B}^{back} /S_{B}^{for}$ (red line with circles), $S_{B}^{up} /S_{A}^{up}$(blue line with triangles), and $S_{A}^{back} /S_{A}^{for}$ (green line with stars) as a function of the wavelength are depicted (**d**), while $\sigma_{sct}^{A}$(red line with squares),$\sigma_{sct}^{B}$ (blue line with triangles) and $\sigma_{ext}$ (black dashed line) are plotted in (**e**). The normalized squared electric field with the gain diagram, when excited at a direction illustrated by yellow arrows, for scenarios 1 and 2 are displayed in (**c**), (**f**), respectively.

For scenario 1, the matchstick with *d*_*rod*_ = 0.33 μm, *d*_*sph*_ = 0.46 μm and *L* = 1.32 μm presents the best performance. Figure 6a shows $S_{A}^{for} /S_{A}^{back}$ (red line with circles), $S_{B}^{up} /S_{A}^{up}$ (blue line with triangles) $S_{B}^{for} /S_{B}^{back}$ (green line with stars) as a function of the wavelength in the visible regime. When excited from side A, the average probability of photon scattering to side B is 22 times higher than backscattering to side A, with a maximum ratio of 46. Consequently, in scenario 1 where the external source of noise photons is located on side A, more photons are forward scattered within the cloud, resulting in increased noise in the image observed from side B. Conversely, when excited from the opposite side, the contrast between forward and backward scattering diminishes, indicating that more photons from side B are reflected within the cloud, contributing to the image degradation on side B while maintaining the image quality on side A. Moreover, there is no significant asymmetry when the cloud is excited from a source atop the cloud ($S_{B}^{up} /S_{A}^{up} \sim 1$). This suggests that as the primary source transition from normal incidence to atop the cloud, the particle loses efficiency in steering the photons to the B side, deteriorating the asymmetrical imaging performance.

Furthermore, the extinction coefficient ($\sigma_{ext}$) and the scattering cross-section when excited from A side ($\sigma_{sct}^{A}$) and B side ($\sigma_{sct}^{B}$) as a function of the wavelength are presented in Fig. 6b. As explained, the extinction coefficient is the same for both sides, resulting in an equal number of photons (power) ballistically forming the target image on both sides of the cloud. Nonetheless, the higher $\sigma_{sct}^{A}$ compared to $\sigma_{sct}^{B}$ indicates that photons arriving from side A are more likely to be scattered within the cloud, thereby increasing the probability of image degradation on side B. Conversely, when arriving from side B, photons are more likely to be absorbed within the cloud, resulting in fewer noise photons on side A, which aligns with the primary objective. To corroborate the particle’s capability to steer photons, the squared normalized scattered electric field $\left| {\mathbf{E}} \right|^{2}$ at *λ* = 590 nm (depicted in Fig. 6c) when excited from A side shows a higher intensity in the front of the matchstick. The higher intensity represents a higher probability of the photon getting forward scattered. Moreover, *G* of the matchstick, presented as an inset in Fig. 6c, has highly directional scattering with negligible sidelobes and backscattering.

For scenario 2, the half-coated sphere with *d*_*cap*_ = 179 nm and *d*_*sph*_ = 35 nm exhibits better performance compared with the matchstick. Figure 6d shows $S_{B}^{back} /S_{B}^{for}$ (red with squares), $S_{B}^{up} /S_{A}^{up}$ (blue with triangles) and $S_{A}^{back} /S_{A}^{for}$ as a function of the wavelength in the visible regime, and Fig. 6e displays $\sigma_{sct}^{A}$(red line with squares), $\sigma_{sct}^{B}$ (blue line with triangles) and $\sigma_{ext}$ (black dashed line). The high value of $S_{B}^{back} /S_{B}^{for}$ indicates that the optimized particle configuration tends to route noise photons from *I*_*0*_ to camera B, surpassing the directing to camera A by up to 4.3 times (at λ = 590 nm) when excited from the B side. Furthermore, the significant value of $S_{B}^{up} /S_{A}^{up}$ at the same wavelength indicates that as the primary source transitions from perpendicular incidence to above the cloud, the cloud remains capable of steering photons toward side B, still improving the performance. Notably, both $S_{B}^{back} /S_{B}^{for}$ and $S_{B}^{up} /S_{A}^{up}$ exceed 1 in the visible range, indicating a higher probability of more noise in the image captured by camera B compared to camera A.

Another important aspect is that the minimum of $\sigma_{ext}$ occurs at the same λ as the maximum of $S_{B}^{back} /S_{B}^{for}$ and $S_{B}^{up} /S_{A}^{up}$. Lower $\sigma_{ext}$ implies higher transmission through the cloud, effectively rendering the cloud as a filter for this specific wavelength. Consequently, the wavelength where more photons from the target pass across the cloud is the same as the maximum of $S_{B}^{back} /S_{B}^{for}$ and $S_{B}^{up} /S_{A}^{up}$, resulting in an enhanced contrast difference between the images observed from both sides. The near-field and gain diagrams depicted in Fig. 6f illustrate that most photons are reflected by the particle at this wavelength, with a smaller portion scattered forward. Therefore, although the cloud routes more noise to camera B than to camera A, both images experience degradation, emphasizing the necessity of carefully designing the cloud properties to detect the target on camera A while hampering camera B. After designing the smoke’s particles, we now analyze its impact on the imaging system when looking at a target on opposite cloud sides.

## Imaging results

To evaluate the performance of the optimized particles, we use the proposed Monte Carlo approach to calculate the Point-source-function (PSF) and Modulation Transfer Function (MTF) for the two scenarios considering the external illumination source and highly asymmetric particles. Furthermore, we evaluate *C*_*A*_, *C*_*ratio*_ and the image formed on both sides using the proposed approach. We consider a symmetrical situation *(L* = *L*_*A*_ = *L*_*B*_) to study only the impact of the Janus particle cloud on the *C*_*ratio*_, disregarding any asymmetric imaging due to the proximity of the cloud to the target or the observer (as seen in Supplementary information). Moreover, we consider both sides of the cloud to present the same power (*P*_*A*_ = *P*_*B*_) and noise photons (*N*_*A*_ = *N*_*B*_). Cameras A and B are simulated with NA = 0.4, 4 Mpixels, and an aperture of L/10,000 (e.g., 3 mm for *L* = 30 m). For the cloud parameters, we set *L*_*cloud*_ = *L*/50, and the cloud particle density *N* is chosen so the transmittance $T = {\text{exp}}\left( { - N\sigma_{ext} L_{cloud} } \right)$ equals to 80%, 60%, 40% and 10%. Our study considers different primary sources of illumination angle (*α*_*I*_) since these parameters can change during the day, and power *I*_*0*_ = 18*P*_*A*_ = 18* PB*. It is important to mention that the illumination source light up the target and the cloud, but only a fraction of the photons from the target are routed toward the cameras, and the number of photons reduces as *L* increases (for more details on this number, see Supplementary information). We divide this section into two parts: first, we consider that all Janus particles are aligned inside the cloud (*σ*_*θ*_ = *σ*_*φ*_ = 0º), and second, we consider misaligned particles.

### Fully aligned particles inside the cloud

As explained, the power of the primary illumination source can vary significantly across different scenarios, such as daytime, nighttime, the presence of clouds, shadows, and the distance between the observer and the target, among other factors. This study considers fully aligned particles within the cloud and examines scenarios where *I*_*0*_ = 18*P*_*A*_*, N*_*A*_ = *N*_*B*_ = 0.5*P*_*A*_ = 0.5* PB*_*,*_ and α_i_ ranges from -90º to 90º. For scenario 1 (*α*_*I*_ < 0), we utilize the optimized matchstick particle, while for scenario 2 (*α*_*I*_ > 0), we employ the coated silica particles.

**Figure 7:**
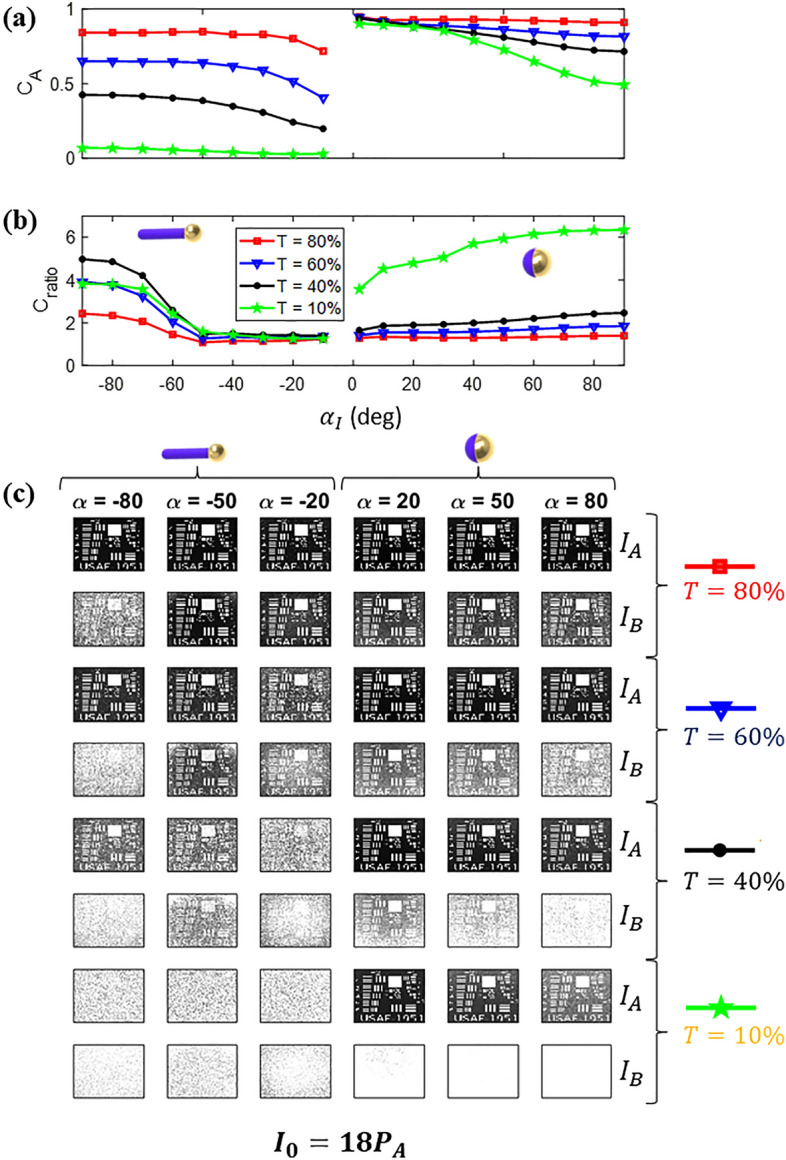
Influence of *α*_*I0*_ on *C*_*A*_ (**a**) and *C*_*ratio*_ (**b**) with varying cloud transmittance (*T*) values: 80% (red squares), 60% (blue triangles), 40% (black circles), and 10% (green stars). Panel (**c**) displays the corresponding images seen through the cloud on Side A (*I*_*A*_—top row) and Side B (*I*_*B*_—bottom row) for different cloud transmittances (80%, 60%, 40%, and 10%) and incident angles (*α*_*I0*_ =  ± 80º, ± 50º, ± 20º).

Figures 7a and b illustrate *C*_*A*_ and *C*_*ratio*_, respectively, of the images observed throughout the cloud, considering *T* values of 80% (red squares), 60% (blue triangles), 40% (black circles) and 10% (green stars). As depicted in Fig. 7a, the image quality seen from side A deteriorates (*C*_*A*_ decreases) as the particle density increases (*T* decreases) since the number of ballistic photons passing through the cloud decrease while the number of scattered noise photons reflected and/or transmitted increases. Moreover, the matchstick's strong directivity combined with its low absorption leads to a significant amount of photons being scattered from *P*_*B*_ and *N*_*B*_ to the image formed on side A, resulting in a noisier image as *T* decreases. Therefore, increasing *I*_*0*_ or changing *α*_*I*_ does not significantly impact *C*_*A*_ as seen in Fig. 7a, since the primary source of noise photons are *P*_*B*_ and *N*_*B*_. On the other hand, the primary source of noise on A side in scenario 2 (Janus sphere) is *I*_*0*_, as evidenced by the decrease in the number of noise photons as $\alpha_{I} \to 0^{ - }$ (see Fig. 6d), and, consequently, the increase in *C*_*A*_.

Even though the cloud degrades the image seen from side A, the image observed from side B experience an even more significant deterioration since the particle optimization favors the cloud for routing the illumination source toward side B, as indicated by *C*_*ratio*_ > 1 in Fig. 7b. This is attributed to the increasing number of noise photons in image B as the transmittance (*T*) decreases, leading to a higher *C*_*ratio*_. However, in scenario 1, the performance of the cloud for asymmetric imaging decreases as the external illumination source transitions *α*_*I*_ from -90º to 0º because the matchstick is optimized only for normal incidence (*α*_*I*_ = − 90º). As shown in Fig. 6a, the low $S_{B}^{up} /S_{A}^{up}$ ratio indicates that the radiation pattern of the matchstick particles is notably directive for *α*_*I*_ = − 90º, but the cloud fails to route more photons to side B when *α*_*I*_ > − 60º. Therefore, *C*_*ratio*_ becomes close to 1 regardless of *T*. In contrast, the coated sphere in scenario 2 exhibits a larger $S_{B}^{up} /S_{A}^{up}$ compared to $S_{B}^{back} /S_{A}^{for}$ leading to a better performance in terms of *C*_*A*_ and *C*_*ratio*_ as $\alpha_{I} \to 0^{ + }$.

To gain a better understanding of the implications of what reducing the contrast represents, Fig. 7c show the image seen through the cloud on side A (*I*_*A*_—top line) and side B (*I*_*B*_—bottom lines) for different cloud transmittances (80%, 60%, 40%, and 10%) and incident angles (α_i_ =  ± 80º, ± 50º, ± 20º). For both scenarios, it is possible to achieve a reasonable contrast ratio (*C*_*ratio*_ > 2) using specific particle density, except for − 60º < *α*_*I*_ < − 10º. This means it would be harder to identify a target seen from side B (worse image) than from side A (better images)—which aligns with the goal of this study. However, even though a high *C*_*ratio*_ is attained, the image quality seen from A side also reduces as *T* diminishes, as evident from examining *I*_*A*_ and *I*_*B*_ in Fig. 7c. Moreover, there is minimal difference between *I*_*A*_ and *I*_*B*_ (*C*_*ratio*_ ~ *1*) for α_I_ = − 50º and − 20º, and both images degrade as *T* decreases, rendering the images equally unidentifiable. In conclusion, while increasing *N* leads to a high *C*_*ratio*_, it may significantly deteriorate the image seen on A to the point where the target cannot be identified from either side. This characteristic highlights the importance of controlling the particle density concerning the external illumination power to achieve optimal performance, as increasing the *C*_*ratio*_ comes with the cost of *C*_*A*_.

### Misalignmed particles inside the cloud

In this study phase, we investigate the effects of misalignments within the cloud, which may arise due to various environmental factors and mechanical forces acting against the alignment system. These misalignments can be triggered by turbulent atmospheric conditions, wind patterns, cloud dynamics, and other external disturbances, causing the particles to deviate from their ideal alignment positions. Such real-world scenarios can lead to diverse misalignment patterns, where the ($\theta , \phi$) angles follow a normal distribution with variances ($\sigma_{\theta } , \sigma_{\varphi }$). Understanding and quantifying the impact of these misalignments on cloud performance is critical for optimizing asymmetric imaging capabilities and ensuring robustness under challenging conditions.

**Figure 8:**
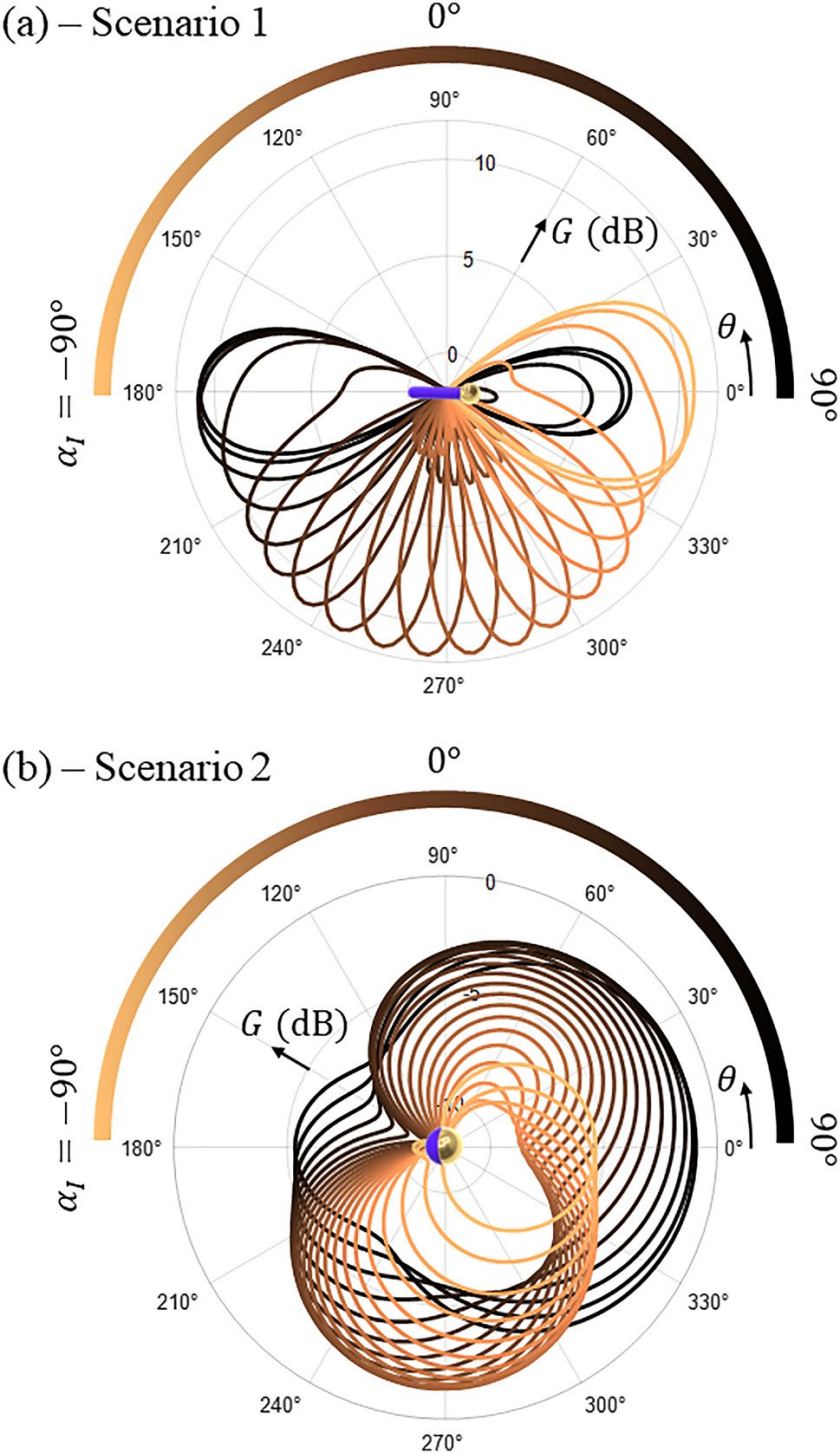
Gain ($G\left( {\theta , \phi = 0} \right)$) of optimized matchstick (**a**) and Janus particle (**b**) as a function of incident angle (*α*_*I*_). As* α*_*I*_ transitions from − 90° (side A) to 90° (side B), the curves become darker. To help the comprehension, the colorbar is curved to represent the direction where incident photons arrive to illuminate the particle.

To gain insights into the impact of misalignments on the imaging system, we first analyze the gain ($G\left( {\theta , \phi = 0} \right)$) of the particles with different incident angles *α*_*I*_. These gain curves are depicted in Fig. 8a and b for scenarios 1 and 2, respectively. In our plots, the gain curves become darker as *α*_*I*_ transitions from − 90° (side A) to 90° (side B).

The analysis of ($G\left( {\theta , \phi = 0} \right)$) for both scenarios reveals significant variations of the phase function with different incident angles, underscoring the necessity of employing the Monte Carlo approach that considers asymmetric particles to predict the cloud imaging system accurately. This nuanced behavior would not be perceptible using regular homogeneous Monte Carlo approaches. For scenario 1, we observe that $G\left( {\theta , \phi = 0} \right)$ exhibits strong directivity for *α*_*I*_ = − 90°, with minimal reflection. However, as *α*_*I*_ increases, side lobes start to emerge on the gain curves, leading to higher reflection. The pronounced directivity implies that fully aligned particles can effectively route photons to side B only when *α*_*I*_ is close to − 90°. Nonetheless, the presence of side lobes generated by misaligned particles could be beneficial for increasing the contrast ratio at higher angles. In this sense, the additional side lobes can redirect photons to side B, enhancing the imaging performance for incident angles where the aligned matchstick fails to achieve high contrast (−60º < *α*_*I*_ < −10º).

In contrast, for scenario 2 (Janus sphere), the directivity is lower, and the particle exhibits a higher reflection for *α*_*I*_ = 90°, accompanied by a lower transmission. As the external illumination angle transitions to *α*_*I*_ = 0^+^, the reflection towards side B decreases, but the transmission towards side A is more impacted, resulting in an improved *C*_*ratio*_. Notably, the Janus sphere also presents a high transmission/reflection ratio for *α*_*I*_ = − 90°, suggesting that the particle could perform in scenario 1 as well, albeit with lower efficiency compared to the optimized matchstick. In this context, specific misalignments could aid in increasing the cloud’s overall performance, especially for *α*_*I*_ = 0^+^.

**Figure 9:**
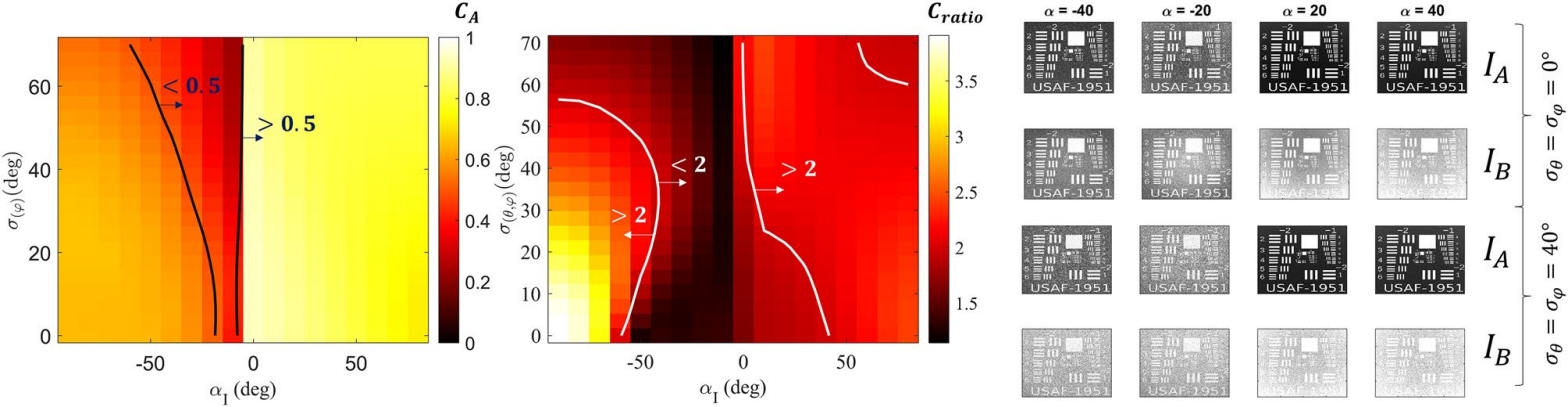
(**a**) and (**b**) present *C*_*A*_ and *C*_*ratio*_, respectively, of images observed through the cloud for scenarios 1 and 2 varying incident angles (*α*_*I*_) and misalignment levels ($\sigma_{\theta } , \sigma_{\varphi }$) (**b**). The black lines in (**a**) divide the regions where the image on the A side presents good contrast (C_A_ > 0.5), and the white lines in (b) divide the regions where the system presents a good contrast ratio (C_ratio_ > 2). The panel in (**c**) shows *I*_*A*_ and *I*_*B*_ for $\alpha_{I} = \pm 40^\circ$ and $\pm 20^\circ$ considering $\sigma_{\theta } = \sigma_{\varphi } =$ 0º (aligned) and 40º (optimum misalignment).

Having analyzed $G$ upon different incident angles (Fig. 8), we now turn our attention to the impact of misalignments within the cloud on the contrast and image quality. For this purpose, we show the results for *C*_*A*_ and *C*_*ratio*_ in Fig. 9a and Fig. 9b, respectively, considering various levels of misalignments ($\sigma_{\theta } , \sigma_{\varphi }$) across both scenarios. The cloud transmission (*T*) is set to 60% and 40% for scenarios 1 and 2, respectively, as this level allows for the generation of *C*_*ratio*_ > 2, as observed in Fig. 7. The black lines in Fig. 9a correspond to *C*_*A*_ = 0.5, indicating the critical threshold for assessing acceptable image quality.

As demonstrated in Fig. 9a, *C*_*A*_ gradually decreases in scenario 1 as we increase the misalignment levels ($\sigma_{\theta } , \sigma_{\varphi }$). The reduction is explained by the fact that as the matchstick gets misaligned, the side lobes and reflection increase (see Fig. 8), rendering more noise photons towards A side. Moreover, the phenomenon leads to a reduction of noise photons routed to B side for *α*_*I*_ < − 60º, resulting better image on B side and a consequent reduction of *C*_*ratio,*_ as seen in Fig. 9b. On the other hand, the side lobes produced by misalignments that reduce *C*_*ratio*_* α*_*I*_ < − 60º help increase *C*_*ratio*_ for* α*_*I*_ > − 60º in scenario 1. When fully aligned, the matchstick is highly directive and fails to route noise photons to B side when *α*_*I*_ > − 60º. However, when we introduce misalignments, part of the matchstick starts routing photons to B side due to their side lobes, deteriorating the image seen from B side and increasing *C*_*ratio*_. In this sense, while fully aligned particles can achieve *C*_*ratio*_ > 2 for incident angles *α*_*I*_ > − 60º, the introduction of misalignments with $\sigma_{\theta } = \sigma_{\varphi } = 40^\circ$ extends this range to *α*_*I*_ > − 30º.

For scenario 2, the misalignments have little impact on *C*_*A*_ since the cloud transmission (*T*) remains approximately the same, rendering the same amount of noise photons deteriorating the image on A side. However, the misalignments significantly increase the photon routing towards side B, especially for incident angles close to *α*_*I*_ = 0°, which leads to an improved *C*_*ratio*_. The Janus sphere used in scenario 2 has a higher transmission-to-reflection ratio for *α*_*I*_ = 0°, allowing for better performance in asymmetric imaging.

To further explore the misaligned impacts on asymmetric imaging, Fig. 9c shows a panel with *I*_*A*_ and *I*_*B*_ for $\alpha_{I} = \pm 40^\circ$ and $\pm \;20^\circ$ considering $\sigma_{\theta } = \sigma_{\varphi } =$ 0º (aligned) and 40º (optimum misalignment). For scenario 1, before considering misalignments, the images *I*_*A*_ and *I*_*B*_ were nearly identical for α_I_ = − 40º and − 20º. However, with the introduction of misalignments, *C*_*ratio*_ improves, causing *I*_*A*_ to exhibit much higher image quality compared to *I*_*B*_ for α_I_ = − 40º. Conversely, for α_I_ = − 20º, both images still demonstrate degradation, and misalignments do not appear to contribute significantly to asymmetric imaging. For scenario 2, misalignments play a role in enhancing *C*_*ratio*_ for both *α*_*I*_ = 40º and 20º. In this sense,* I*_*B*_ exhibits slightly worse identification potential as we introduce misalignments, aligning with the study’s primary objective.

These results emphasize the importance of considering misalignments within the cloud when optimizing particle design for asymmetric imaging. Incorporating misalignments can lead to enhanced/reduced contrast ratios, altering the range of incident angles with *C*_*ratio*_ > 2 for specific scenarios. Moreover, it shows that the directivity of the particles plays a crucial role in photon redirection and image formation, highlighting to importance of the proper Monte Carlo approach to simulate asymmetric particles to optimize the cloud's performance.

## Probability of detection and identification

Investigating the effects of aligned and misaligned particles within the cloud has provided valuable insights into their influence on image quality and contrast ratio in various scenarios. Our analyses have revealed image quality and contrast changes due to misalignments, highlighting the significance of accurately calculating the PD and PID in such cloud environments. Accurate PD and PID calculation is crucial for assessing the efficacy of surveillance systems and making informed decisions regarding deployment strategies. To this end, we use Eqs. (20), (21) considering *V*_*50*_ = 5 (2), a reasonable value for PID (PD) a 30 cm size face from a distance *d* = *R*_*tgt*_. It is worth noticing that the MTF is calculated as a function of *k*_*i*_ and *k*_*j*_, and the target size can be adjusted by manipulating its spatial frequencies $\xi \;{\text{and}} \;\psi$ accordingly. For CTF_eye_, we employ^83^ considering the luminescence of 50 kcd/m^2^ altered by the cloud's transmittance. Moreover, to assess the asymmetric imaging in the cloud, we define a reasonable performance when it is possible to identify the target more than 50% of the time on side A (PID_A_ > 50%) while the probability of detecting a target on side B is less than 50% (PD_B_ < 50%).

**Figure 10:**
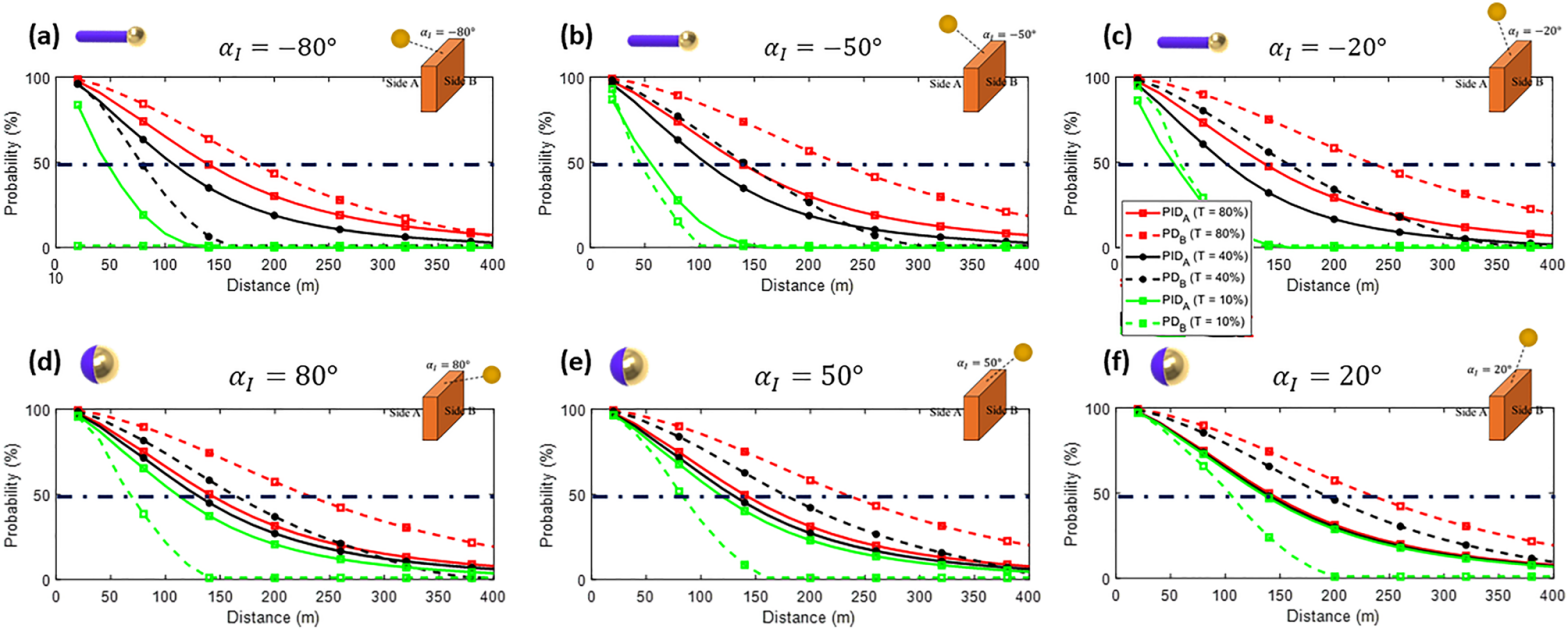
PID_A_ (solid lines) and PD_B_ (dashed lines) of a target considering *α*_*I*_ = − 80º (**a**), − 50º (**b**), − 20º (**c**), 20º (**d**), 50º (**e**), 80º (**f**) for *T* values of 80% (red squares), 40% (black circles) and 10% (green stars).

Figure 10a–f show PID_A_ (solid lines) and PD_B_ (dashed lines) considering *α*_*I*_ = − 80º (a), − 50º (b), − 20º (c), 20º (d), 50º (e), 80º (f) for *T* values of 80% (red squares), 40% (black circles) and 10% (green stars). The dash-dot lines represent the 50% line, and we consider fully aligned particles inside the cloud. As expected, both PID_A_ and PD_B_ decrease as the distance from the target increases due to the reduction in the target-formed image size on the eye. Furthermore, the concealment difference between both sides increases as *T* is reduced. For scenario 1, the cloud's performance improves as *α*_*I*_ tends to -90º as expected based on *C*_*ratio*_ calculation. In scenario 2, a favorable contrast can be achieved for *T* = 10% in all scenarios, where PID_A_ can exceed 50% while PD_B_ is maintained below 50% at certain distances. In summary, the PID_A_ > 50% and PD_B_ < 50% metric is consistently achieved for *T* = 10%, except for 0º < *α*_*I*_ < − 50º. In this sense, we can identify a target more than 50% of the time from side A (PID_A_ > 50%), while the same target is not even detected more than 50% of the time from side B (PD_B_ < 50%), which is this manuscript's main goal. It is worth emphasizing that the detection/identification tasks analyzed here consider the performance of the naked eye. Although advanced imaging techniques can be employed to improve the contrast of the images, they are only feasible if the image is captured and further processed by an electronic system, which is out of the scope of our study at this time. Additional graphs comparing the PIDs and PDs can be found in the Supplementary information. Overall, this study demonstrates the efficacy of the proposed cloud engineered with Janus particles, enabling the generation of asymmetric imaging with simultaneous concealment from one side and clear visualization from the other.

## Conclusion

In this manuscript, we present an approach for engineering a single obscurant smoke comprised of Janus particles capable of providing clear visualization from one side while distorting and concealing the image on the opposite side. Our model effectively examines the cloud's influence on image contrast and contrast ratio, offering valuable insights for achieving asymmetric imaging. By expanding the scattering properties of a single particle to model the cloud behavior, we optimize the contrast ratio by tailoring the particle design. The model shows that two different optimization functions are attained depending on the primary source of photons: behind the cloud (scenario 1) and in front of the cloud (scenario 2). In this sense, we develop two distinct nanoparticles, one for each scenario, a matchstick for scenario 1, and a half-coated silica sphere for scenario 2. These engineered particles route noise photons to a single cloud side, effectively obscuring the target from view on the other side. After the particle optimization, we described a Monte Carlo approach that can calculate the PSF, MTF, and contrast of an image seen from both sides of a cloud composed of highly asymmetric and inhomogeneous scatterers. Our approach accommodates the possibility of misaligned particles within the cloud and incorporates random noise photons from an external illumination source. By carefully controlling the particle density within the smoke, we achieve a high contrast ratio (*C*_*ratio*_ > 2) for *α*_*I*_ < − 60º (scenario 1) and *α*_*I*_ > 0º (scenario 2) without significantly compromising image quality on side A. Additionally, we investigate the impact of misaligned particles within the cloud, revealing their potential to enhance contrast ratio in specific conditions. Misalignments allow for extended operation ranges to *α*_*I*_ > − 30º when using $\sigma_{\theta } = \sigma_{\varphi } = 40^\circ$. Finally, the optimized Janus particle obscurants cloud yields a target identification task with a success rate exceeding 50% from side A, while a detection task has a successful success rate of less than 50% from side B at certain distances. This research opens new possibilities for modern obscurant design and imaging systems for highly asymmetric and inhomogeneous particles.

### Supplementary Information

Supplementary Information.

## Data Availability

The datasets used and/or analysed during the current study available from the corresponding author on reasonable request.
