# Supplementary material for: Asymmetric imaging through engineered Janus particle obscurants using a Monte Carlo approach for highly asymmetric scattering media
Source: Sci Rep. 2024 Feb 15;14:3850. doi: 10.1038/s41598-024-54035-7 (PMC10869813; doi:10.1038/s41598-024-54035-7)
Supplement: Supplementary file 1 — Supplementary Information. [file 41598_2024_54035_MOESM1_ESM.pdf]

Supporting Information for:

Asymmetric imaging through engineered Janus particle obscurants using a Monte Carlo approach for highly asymmetric scattering media

Achiles F. da Mota[1,2], Mohammad Mojtaba Sadafi[1], Hossein Mosallaei[1]

[1] Department of Electrical Engineering, Northeastern University - 02115, Boston, MA.

[2] Department of Electrical Engineering, University of Brasília (UnB) – Zip code: 70910-900, Brasilia, Brazil

Keywords: asymmetric imaging, Janus nanoparticles, obscurants smoke, Surveillance Reconnaissance Systems, Monte Carlo ray-tracing, scattering theory.

1. Modeling the particle using discrete dipole approximation

To calculate the scattering properties of the particles inside the cloud, we resort to the well-known Discrete Dipole Approximation (DDA) [1], [2], [3]. In this approach, the scatter is discretized into *N* lattice points with size d_x, and the *j*th element presents polarizability tensor $\boldsymbol{\alpha}_\mathbf{j} = \mathbf{I}\left(3\varepsilon_0 d_x^3(\varepsilon_s - \varepsilon_m)/(\varepsilon_s + 2\varepsilon_m)\right)$, where $\varepsilon_s, \varepsilon_m$, ε_0 are lattice, surrounding medium and vacuum permittivity, respectively, as shown in Figure S2. Using the framework of the DDA, the dipole moment of the *j*th nano-antenna cell $\mathbf{p_j}$ is given by

$$\mathbf{p_j} = \boldsymbol{\alpha}_\mathbf{j}\mathbf{E_{inc}}(\mathbf{r_j}) + \sum_{\mathbf{n=1}}^{N} \mathbf{G_{jn}}\mathbf{p_n}, \tag{1.1}$$

where $\mathbf{E_{inc}}(\mathbf{r_j})$ is the incident electric field at the *j*th lattice position $\mathbf{r_j}$, $\mathbf{G_{jn}}$ is the Green-tensor from the *j*th to the *n*th element, given by,

$$\mathbf{G_{jn}} = \omega^2\mu_0\left(\mathbf{I} - \frac{\nabla\nabla}{k^2}\right)\frac{e^{\mathrm{j}k(\mathbf{r_j}-\mathbf{r_n})}}{4\pi|\mathbf{r_j}-\mathbf{r_n}|}. \tag{1.2}$$

When equation 2.2 is expressed for the *N* lattice points, it becomes a linear system solved using numerical approaches to compute $\mathbf{p_j}$, such as the generalized minimal residual method (GMRS), or the quasi-minimal residual method (QMRM). When the particles are inside the cloud, the scattered photon could collide with the particle from any incident direction $(\theta_i,\ \varphi_i)$, therefore, to compute for all the possible situations, we compute $\mathbf{p_j}(\theta_i,\ \varphi_i)$ for all possible incidences, given by

$$\mathbf{E_{inc}}(\mathbf{r_j}, \theta_i,\ \varphi_i) = \mathbf{E_0}\mathbf{e}^{\mathbf{jk_{inc}}(\theta_i,\ \varphi_i)\mathbf{r_j}}, \begin{cases} 0 < \theta_i < \pi \\ 0 < \varphi_i < 2\pi \end{cases}. \tag{1.3}$$

Note that $\mathbf{E_0}$ is chosen so the incident wave has power equally distributed between p- and s-polarization. After solving the linear system, the extinction cross-section $(\sigma_{ext}(\theta_i,\ \varphi_i))$ is calculated as,

$$\sigma_{ext}(\theta_i,\ \varphi_i) = \frac{W_{tot}}{I_{inc}} = \omega Z_0 \mathrm{Im}\left\{\sum_{j=1}^{N_{dip}} \mathbf{E_{inc}}(\mathbf{r_j}, \theta_i,\ \varphi_i)\cdot \mathbf{p_j}(\theta_i,\ \varphi_i)^*\right\}. \tag{1.4}$$

where W_{tot} is the total power dissipated by the particle, Z_0 is the background wave impedance, ω is the angular frequency and I_{inc} is the intensity of the incident wave ($I_{inc} = 1/2Z_0$). Note that the extinction coefficient of a cloud is given by $\mu_{ext}(\theta_i,\ \varphi_i) = \sigma_{ext}(\theta_i,\ \varphi_i)\ \rho_{cloud}$, where ρ_{cloud} is the particle density. The radiation intensity $U(\theta_i,\ \varphi_i, \theta, \varphi)$ for a given incident wave is

$$U(\theta_i,\ \varphi_i, \theta, \varphi) = \frac{1}{2Z_0}\left|\frac{\omega^2\mu_0}{4\pi}\sum_j^{N_{dip}} e^{-\mathrm{j}k_0 r_j}\begin{bmatrix} 0 & 0 & 0 \\ sin(\varphi) & -cos(\varphi) & 0 \\ cos(\varphi)\,cos(\theta) & sin(\varphi)\,cos(\theta) & sin(\theta) \end{bmatrix}\mathbf{p_j}(\theta_i,\ \varphi_i)\right|^2, \tag{1.5}$$

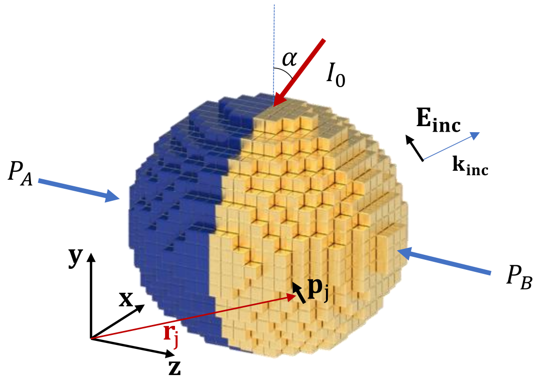

Figure S1- Discretized Particles for Direct Dipole Approximation method.

and the total radiated power ($W_{rad}(\theta_i,\ \varphi_i)$) can be calculated using,

$$W_{rad}(\theta_i,\ \varphi_i) = \int_0^{\pi} d\theta \int_0^{2\pi} U(\theta_i,\ \varphi_i, \theta, \varphi)\, sin\, \theta\, d\varphi. \tag{1.6}$$

With $W_{rad}(\theta_i,\ \varphi_i)$, we can calculate the scattering cross-section of the particle ($\sigma_{sct}(\theta_i,\ \varphi_i)$) and the directivity ($D(\theta_i,\ \varphi_i, \theta, \varphi)$), respectively, as

$$\sigma_{sct}(\theta_i,\ \varphi_i) = \frac{W_{rad}(\theta_i, \varphi_i)}{I_{inc}}, \tag{1.7}$$

$$D(\theta_i,\ \varphi_i, \theta, \varphi) = 4\pi \frac{U(\theta_i, \varphi_i, \theta, \varphi)}{W_{rad}(\theta_i, \varphi_i)}, \tag{1.8}$$

We can think that the scatter behaves as an antenna, where it redirects an incoming photon in the direction $\theta_i,\ \varphi_i$ to a new direction θ, φ with efficiency $\eta = \sigma_{sct}(\theta_i,\ \varphi_i)/\sigma_{ext}(\theta_i,\ \varphi_i)$ – part of the photons are absorbed and dissipated by the particle. Moreover, the directivity gives information on the new photon direction since it behaves as a probability distribution function. In this sense, we can calculate the probability of a photon being forward- or backward-scattered, given its incoming directions, as seen in Figure S2. Assuming a photon arriving from side A (B), the total forward ($\sigma_{A(B)}^{for}$) and backward ($\sigma_{A(B)}^{back}$) scatterings are calculated as

$$\sigma_{A(B)}^{for}(\theta_i,\ \varphi_i) = \int_{0(3\pi/4)}^{\frac{\pi}{4}(\pi)} d\theta \int_0^{2\pi} \frac{U(\theta_i, \varphi_i, \theta, \varphi)}{I_{inc}} \sin\theta\, \mathrm{d}\varphi, \tag{1.9}$$

$$\sigma_{A(B)}^{back}(\theta_i,\ \varphi_i) = \int_{\frac{3\pi}{4}(0)}^{\pi(\frac{\pi}{4})} d\theta \int_0^{2\pi} \frac{U(\theta_i, \varphi_i, \theta, \varphi)}{I_{inc}} \sin\theta\, \mathrm{d}\varphi. \tag{1.10}$$

Finally, the total forward ($\sigma_{A(B)}^{for}$) and backward ($\sigma_{A(B)}^{back}$) scattering from a photon arriving from an external illumination source with angle α is given by,

$$\sigma_{A(B)}^{up}(\theta_i,\ \varphi_i) = \int_{\frac{3\pi}{4}(0)}^{\pi(\frac{\pi}{4})} d\theta \int_0^{2\pi} \frac{U\left(\frac{\pi}{2}+\alpha, \pi, \theta, \varphi\right)}{I_{inc}} \sin\theta\, \mathrm{d}\varphi. \tag{1.11}$$

Up to this point, we have calculated the properties of a single particle. However, the single-particle properties are related to the cloud properties by $S_{A(B)}^{for} = K_{A(B)}^{for} \sigma_{A(B)}^{for}(\theta_i,\ \varphi_i)$, $S_{A(B)}^{back} = K_{A(B)}^{back} \sigma_{A(B)}^{back}(\theta_i,\ \varphi_i)$ and $S_{A(B)}^{up} = K_{A(B)}^{back} \sigma_{A(B)}^{up}(\theta_i,\ \varphi_i)$, where K is a constant of proportionality dependent on several parameters, including L_{cloud} and ρ.

2. Reciprocity Theorem

To prove the reciprocity, we start by expanding the propagating electric field at the point **r** in terms of electromagnetic multipoles as follows,

$$\mathbf{E}(\mathbf{r}) = \sum_{l=1}^{\infty}\sum_{m=-l}^{l} a_l^m \mathbf{M_l^m}(\mathbf{r}) + b_l^m \mathbf{N_l^m}(\mathbf{r}), \tag{2.1}$$

where m and l are integers corresponding to the multipoles, $\mathbf{M_l^m}$ and $\mathbf{N_l^m}$ are the regular electric and magnetic multipoles, respectively, and a_l^m and b_l^m are the electric and magnetic multipoles moments, respectively. In the manuscript, the particles are excited by a plane wave propagating with an elevation and azimuthal angles (θ_i,φ_i). The electric field of the plane wave can be written by substituting the multipole moments as follows,

$$\mathrm{TM} \Rightarrow \begin{cases} a_l^m = E_0(\mathrm{j}^{l-1})\pi_l^m(\theta_i)\mathrm{e}^{-\mathrm{j}m\phi_i} \\ b_l^m = E_0(\mathrm{j}^{l-1})\tau_l^m(\theta_i)\mathrm{e}^{-\mathrm{j}m\phi_i} \end{cases}, \tag{2.2}$$

$$\mathrm{TE} \Rightarrow \begin{cases} a_l^m = -E_0(\mathrm{j}^{l})\tau_l^m(\theta_i)\mathrm{e}^{-\mathrm{j}m\phi_i} \\ b_l^m = -E_0(\mathrm{j}^{l})\pi_l^m(\theta_i)\mathrm{e}^{-\mathrm{j}m\phi_i} \end{cases}, \tag{2.3}$$

where E_0 is the amplitude of the electric field, TM and TE denote transversal magnetic and transversal electric, and

$$\begin{cases} \pi_l^m(\theta_i) = \sqrt{\dfrac{(2l+1)(l-m)!}{l(l+1)(l+m)!}}\dfrac{m}{\sin\theta_i}P_l^m(\cos\theta_i) \\ \tau_l^m(\theta_i) = \sqrt{\dfrac{(2l+1)(l-m)!}{l(l+1)(l+m)!}}\dfrac{\partial}{\partial\theta}P_l^m(\cos\theta_i) \end{cases}, \tag{2.4}$$

where, $P_l^m(\cos\theta_i)$ is the associated Legendre polynomial. Note in (4), that when the wave arrives from opposites sides, $\pi_l^m(\pi-\theta_i) = (-1)^{l+m}\pi_l^m(\theta_i)$ and $\tau_l^m(\pi-\theta_i) = -(-1)^{l+m}\tau_l^m(\theta_i)$.

To prove the reciprocity theorem, we need to prove that the cloud extinction $\mu_{ext}(\theta_i)$ when $\theta_i = 0°$ is equal to when $\theta_i = 180°$. Note that the particle can be oriented in any direction, holding this as a general proof. The light scattered by the particle when the light arrives from $\theta_i = 0°$ ($\boldsymbol{E}_{sct}^{+}$) and $\theta_i = 180°$ ($\boldsymbol{E}_{sct}^{-}$) are given by,

$$\boldsymbol{E}_{sct}^{\pm}(\mathbf{r}) = \sum_{l=1}^{\infty}\sum_{m=-l}^{l} p_l^{m,\pm}\mathbf{M_l^m}(\mathbf{r}) + q_l^{m,\pm}\mathbf{N_l^m}(\mathbf{r}), \tag{2.5}$$

where $p_l^{m,\pm}$ and $q_l^{m,\pm}$ are the electric and magnetic multipole moments of the scattered wave, respectively. The most common approach to calculate p_l^m and q_l^m is by means of using the T-matrix. Using this technique, the p_l^m and q_l^m can be related to the moments of the incident wave by a matrix multiplication operation, as follows,

$$\begin{bmatrix}\mathbf{P}^+ \\ \mathbf{Q}^+\end{bmatrix} = \begin{bmatrix}\mathbf{AA} & \mathbf{AB} \\ \mathbf{BA} & \mathbf{BB}\end{bmatrix}\begin{bmatrix}\mathbf{A_{inc}}(\theta_i = 0) \\ \mathbf{B_{inc}}(\theta_i = 0)\end{bmatrix}, \tag{2.6}$$

$$\begin{bmatrix}\mathbf{P}^- \\ \mathbf{Q}^-\end{bmatrix} = \begin{bmatrix}\mathbf{AA} & \mathbf{AB} \\ \mathbf{BA} & \mathbf{BB}\end{bmatrix}\begin{bmatrix}\mathbf{A_{inc}}(\theta_i = \pi) \\ \mathbf{B_{inc}}(\theta_i = \pi)\end{bmatrix}, \tag{2.7}$$

where $\mathbf{P}^{\pm}$ and $\mathbf{Q}^{\pm}$ are the truncated multipole moments for scattered field ($l = 1..L$), $\mathbf{A_{inc}}$ and $\mathbf{B_{inc}}$ are the truncated multipole moments of the incident field, and **AA, AB, BA** and **BB** are the matrices that's related the incident field moments with the scattered field moments. Considering a TM incidence (the same procedure can be done for TE), equations (2.6) and (2.7) can be rewritten in its linear form as follows,

$$\begin{cases} p_l^{m,+} = \sum_{l`=1}^{\infty}\sum_{m`=-l}^{l} AA_{m,m`}^{l,l`}a_{l'}^{m'} + AB_{m,m`}^{l,l`}b_{l'}^{m'} \\ q_l^{m,+} = \sum_{l`=1}^{\infty}\sum_{m`=-l}^{l} BA_{m,m`}^{l,l`}a_{l'}^{m'} + BB_{m,m`}^{l,l`}b_{l'}^{m'} \end{cases}, \tag{2.8}$$

$$\begin{cases} p_l^{m,-} = \sum_{l`=1}^{\infty} \sum_{m`=-l}^{l} AA_{m,m`}^{l,l`} a_{l'}^{m'} (-1)^{l'+m'} - AB_{m,m`}^{l,l`} b_{l'}^{m'} (-1)^{l'+m'} \\ q_l^{m,-} = \sum_{l`=1}^{\infty} \sum_{m`=-l}^{l} BA_{m,m`}^{l,l`} a_{l'}^{m'} (-1)^{l'+m'} - BB_{m,m`}^{l,l`} b_{l'}^{m'} (-1)^{l'+m'} \end{cases}. \quad (2.9)$$

Using (2.8) and (2.9), we can calculate the extinction coefficient ($C_{ext}^{\pm}$) for the particle as,

$$C_{ext}^{\pm} = \frac{4\pi}{k_0^2} \mathrm{Re} \left[\sum_{l=1}^{\infty} \sum_{m=-l}^{l} p_l^{m,\pm} a_l^{m*} + q_l^{m,\pm} b_l^{m*} \right] \quad (2.10)$$

By substituting (2.8) and (2.9) into (2.10), we have,

$$C_{ext}^{+} = \frac{4\pi}{k_0^2} \mathrm{Re} \left\{ \sum_{l=1}^{\infty} \sum_{m=-l}^{l} \left[\left(\sum_{l`=1}^{L} \sum_{m`=-l}^{l} AA_{m,m`}^{l,l`} a_{l'}^{m'} + AB_{m,m`}^{l,l`} b_{l'}^{m'} \right) a_l^{m*} + \left(\sum_{l`=1}^{L} \sum_{m`=-l}^{l} BA_{m,m`}^{l,l`} a_{l'}^{m'} + BB_{m,m`}^{l,l`} b_{l'}^{m'} \right) b_l^{m*} \right] \right\} \quad (2.11)$$

$$C_{ext}^{-} = \frac{4\pi}{k_0^2} \mathrm{Re} \left\{ \sum_{l=1}^{\infty} \sum_{m=-l}^{l} \left[\left(\sum_{l`=1}^{L} \sum_{m`=-l}^{l} AA_{m,m`}^{l,l`} a_{l'}^{m'} (-1)^{l'+m'} - AB_{m,m`}^{l,l`} b_{l'}^{m'} (-1)^{l'+m'} \right) a_l^{m*} (-1)^{l+m} + \left(\sum_{l`=1}^{L} \sum_{m`=-l}^{l} BA_{m,m`}^{l,l`} a_{l'}^{m'} (-1)^{l'+m'} - BB_{m,m`}^{l,l`} b_{l'}^{m'} (-1)^{l'+m'} \right) \left(-b_l^{m*} (-1)^{l+m} \right) \right] \right\} \quad (2.12)$$

As a property of the T-matrix, $AA_{m,m`}^{l,l`} = (-1)^{l+m} AA_{m`,m}^{l`,l}$, $BB_{m,m`}^{l,l`} = (-1)^{l+m} BB_{m`,m}^{l`,l}$ and $AB_{m,m`}^{l,l`} = -(-1)^{l+m} BA_{m`,m}^{l`,l}$ [4]. In this sense, (2.12) can be written as

$$C_{ext}^{-} = \frac{4\pi}{k_0^2} \mathrm{Re} \left\{ \sum_{l=1}^{\infty} \sum_{m=-l}^{l} \left[\left(\sum_{l`=1}^{L} \sum_{m`=-l}^{l} AA_{m,m`}^{l,l`} a_{l'}^{m'} + AB_{m,m`}^{l,l`} b_{l'}^{m'} \right) a_l^{m*} + \left(\sum_{l`=1}^{L} \sum_{m`=-l}^{l} BA_{m,m`}^{l,l`} a_{l'}^{m'} + BB_{m,m`}^{l,l`} b_{l'}^{m'} \right) b_l^{m*} \right] \right\} = C_{ext}^{+} \quad (2.13)$$

Since $\mu_{ext}^{\pm} = N C_{ext}^{\pm} L_{cloud}$, where N is the particle density (1/m^3) and L_{cloud} is the cloud length, eq. (2.13) proves that the ballistic attenuation of the cloud when the photon arrives from opposite sides are the same.

3. Sun Position

We have considered the same number of photons for all radiation sources on the Monte Carlo approach. However, some of these powers have different values than others, and here we show the relation between them. Figure S2 shows that the cloud is illuminated by the external source of power (I_0) and by a single pixel on side A (P_A). Considering L_A and L_B as the distance between the target on side A and the observer on side B to the cloud, respectively, and also *NA* as the numerical aperture of the camera used by the observer, the maximum aperture angle (α_{max}) radiated by P_A that can be scattered to B side can be calculated as,

$$\alpha_{max} = tan^{-1}[(L_B/L_A)\, tan(asin(NA))]. \quad (3.1)$$

Note that we consider α_{max} in our Monte Carlo Approach, and all photons are generated to be inside this region. From this, we can calculate the total power radiated by I_0 to the cloud (note that in our approach, I_0 is normalized by the total number of pixels N_{pixels}^2),

$$I_0 = \frac{W_0 A_{cloud}}{N_{pixels}^2} = \frac{W_0 (2L_A \tan \alpha_{max})^2}{N_{pixels}^2} = \frac{W_0 (2L_B \tan NA)^2}{N_{pixels}^2}. \quad (3.2)$$

where W_0 is the power density (W/m^2) of I_0, and $A_{cloud} = (2L_A \tan \alpha_{max})^2$ is the area illumination. The primary illumination source also illuminates P_A, which absolves A_{abso} of the photons and scatterers all photons isotropically. However, we only considered in the Monte Carlo the photons inside going to the illumination area. In this sense, the P_A can be calculated using the relation

$$P_A = W_0 A_{pixel}(1 - A_{abso})\frac{(1-\cos\alpha_{max})}{2} = W_0 \frac{\{2(L_A+L_B)\tan[asin(NA)]\}^2}{N_{pixels}^2}(1 - A_{abso})\frac{(1-\cos\alpha_{max})}{2}. \quad (3.3)$$

where the $A_{pixel} = \frac{\{2(L_A+L_B)\tan[asin(NA)]\}^2}{N_{pixels}^2}$ is the pixel area. Finally, a relation between I_0 and P_A can be obtained as,

$$\frac{I_0}{P_A} = \left(\frac{L_B}{L_A+L_B}\right)^2 \frac{2}{(1-A_{abso})(1-\cos\alpha_{max})}. \quad (3.4)$$

The relation $\frac{I_0}{P_B}$ can be readily obtained by exchanging L_A and L_B on the equations. Figure S3 shows I_0/P_B(blue) and I_0/P_A (red) as a function of the relative distance position of the cloud between side A and B ($L_A/(L_A + L_B)$) and considering $A_{abso} = 0.5$ and NA=0.33 as inside the main manuscript. When the cloud is at the center, the impact of the photons generated by the external illumination is the same on both sides of the cloud, and a factor of 18 needs to be applied. When the cloud moves close to side A ($L_A/(L_A + L_B)$<0.5), I_0/P_A becomes higher than I_0/P_B. This represents that the I_0 sends more noise photons to side B (since the power of I_0/P_A is higher) then to side A. In this sense, positioning the cloud closer to the target on side A would also help increase the contrast ratio, which is the primary goal of the manuscript.

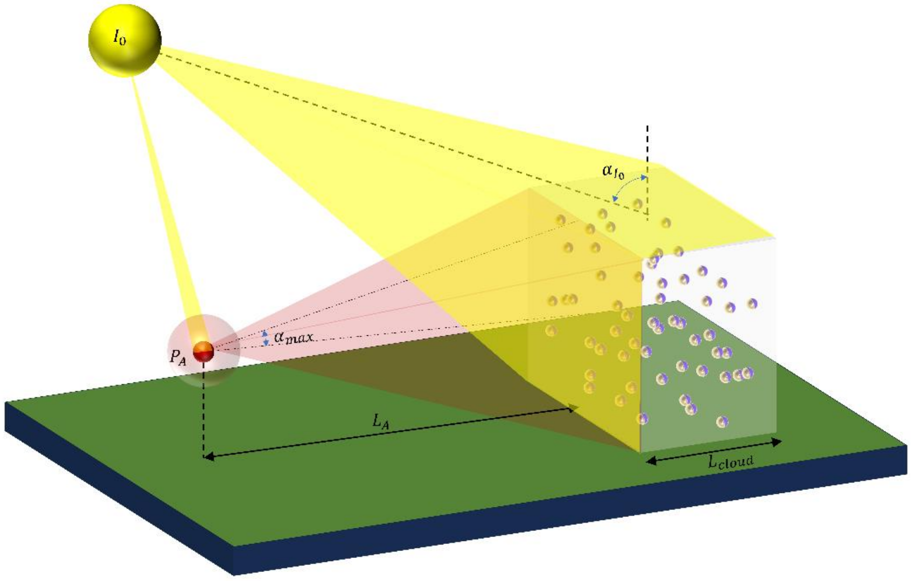

Figure S2 - Cloud being illuminated by the external source of power (I_0) and by a single pixel on side A (P_A). L_A and L_B are the distance between the target on side A and the observer on side B to the cloud, respectively

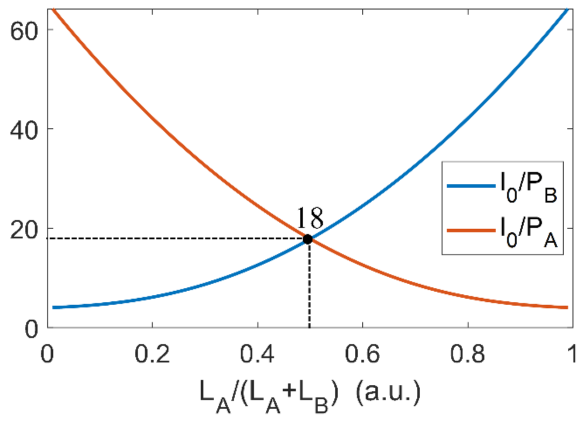

Figure S3 - I_0/P_B(blue) and I_0/P_A (red) as a function of the relative distance position of the cloud between sides A and B ($L_A/(L_A + L_B)$) and considering $A_{abso} = 0.5$ and NA=0.33

4. Probability of detection and identification

In the main manuscript, we explored the Probability of Identification from side A (PID_A) compared to the Probability of Detection from side B (PDB). To enrich the discussion and reinforce the asymmetric imaging through the cloud, we compare PIDs and PDs. The PID comparison is presented in Figures S4 (a-f), where PID_A (solid lines) and PID_B (dashed lines) are calculated considering α_I = -80º (a), -50º (b), -20º (c), 20º (d), 50º (e), 80º (f) for T values of 80% (red squares), 40% (black circles) and 10% (green stars). Figures S5 (a-f) present PD_A (solid lines) and PD_B (dashed lines) are calculated considering α_I = -80º (a), -50º (b), -20º (c), 20º (d), 50º (e), 80º (f) for T values of 80% (red squares), 40% (black circles) and 10% (green stars). Note from Figs. S4 and S5 that the $PID_A(PD_A)$> $PID_B(PD_B)$ for all scenarios (solid lines are always higher than dashed lines), despite T and α_I, emphasizing the cloud capacity to perform asymmetric imaging.

For all PID and PD calculations, we have used the target image shown in Fig. S6.

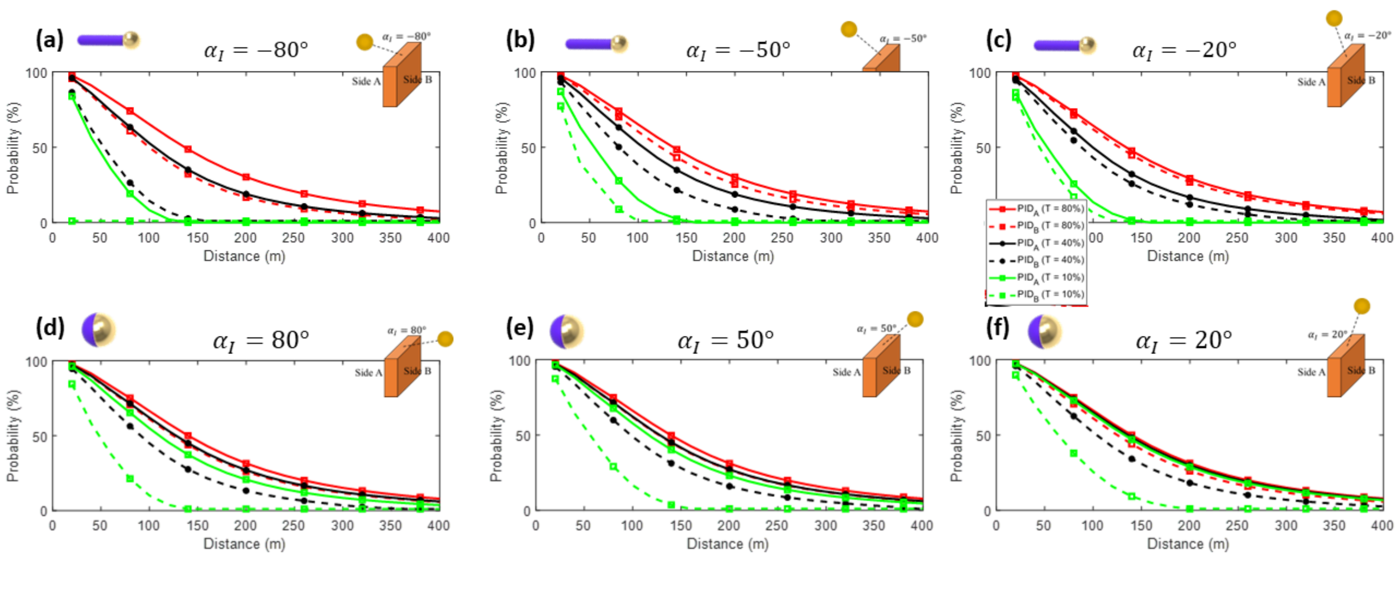

Figure S4 - PID_A (solid lines) and PID_B (dashed lines) of a target considering α_I = -80º (a), -50º (b), -20º (c), 20º (d), 50º (e), 80º (f) for T values of 80% (red squares), 40% (black circles) and 10% (green stars).

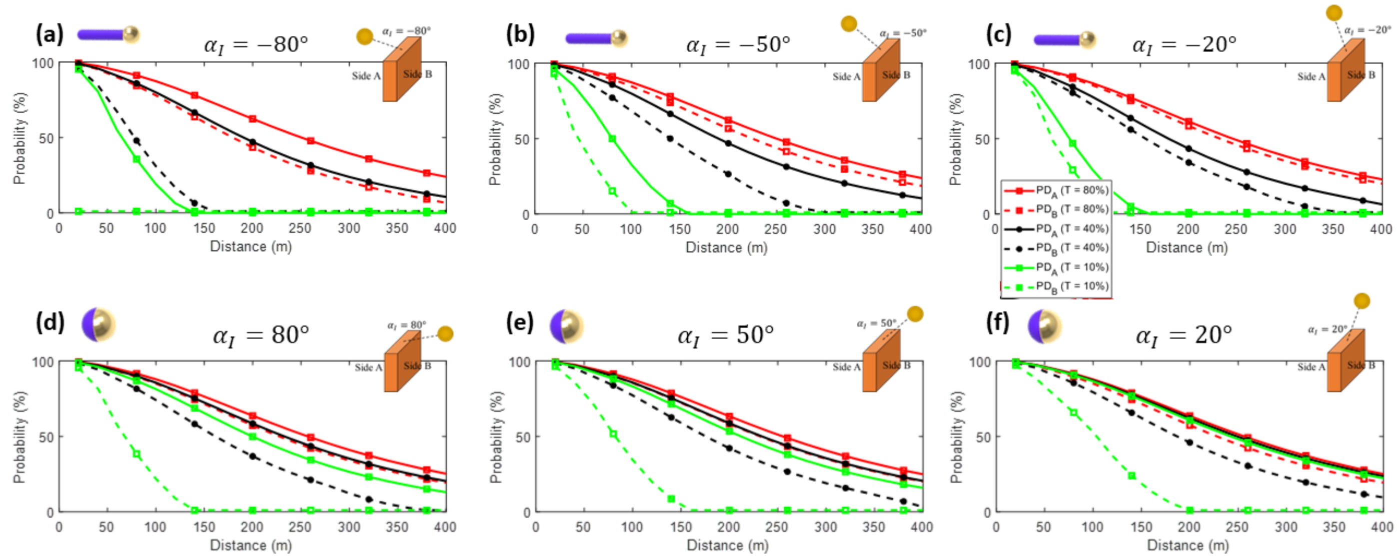

Figure S5 - PD_A (solid lines) and PD_B (dashed lines) of a target considering α_I = -80º (a), -50º (b), -20º (c), 20º (d), 50º (e), 80º (f) for *T* values of 80% (red squares), 60% (blue triangles), 40% (black circles) and 10% (green stars).

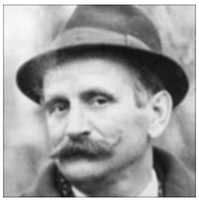

Figure S6 – Target image used for PD/PID calculations.

5. Bibliography

[1] B. T. Draine and P. J. Flatau, “Discrete-dipole approximation for scattering calculations,” 1994.

[2] M. A. Yurkin and A. G. Hoekstra, “The discrete dipole approximation: An overview and recent developments,” *J Quant Spectrosc Radiat Transf*, vol. 106, no. 1–3, pp. 558–589, Jul. 2007, doi: 10.1016/j.jqsrt.2007.01.034.

[3] M. Mahdi Salary, A. Forouzmand, and H. Mosallaei, “Model order reduction of large-scale metasurfaces using a hierarchical dipole approximation,” *ACS Photonics*, vol. 4, no. 1, pp. 63–75, Jan. 2017, doi: 10.1021/acsphotonics.6b00568.

[4] Y. Jing, H. Chu, B. Huang, J. Luo, W. Wang, and Y. Lai, “A deep neural network for general scattering matrix,” *Nanophotonics*, vol. 12, no. 13, pp. 2583–2591, Jun. 2023, doi: 10.1515/nanoph-2022-0770.
